# Astrocytic mitochondria restore neuronal bioenergetics and function in Parkinson disease models

**DOI:** 10.1016/j.isci.2026.118138

**Published:** 2026-11-06

**Authors:** Jitendra Singh, Virendra Tiwari, Alka Sharma, Shubha Shukla

**Affiliations:** 1 Division of Neuroscience and Ageing Biology, CSIR- Central Drug Research Institute, Lucknow, UP 226031, India; 2 Academy of Scientific and Innovative Research (AcSIR), Ghaziabad 201002, India; 3 Department of Pharmacology, GITAM School of Pharmacy, GITAM (Deemed to be University), Hyderabad, Telangana 502329, India

**Keywords:** astrocytes, neurons, mitochondrial therapy, Parkinson’s disease, 6-hydroxydopamine, 6-OHDA, neuroprotection, mitochondrial transfer

## Abstract

Mitochondrial dysfunction contributes to neuronal injury in Parkinson’s disease, and astrocyte-derived mitochondria may offer neuroprotection by restoring neuronal bioenergetics. Here, we investigated the therapeutic potential of astrocyte-derived mitochondria in 6-OHDA models of Parkinson’s disease. Using mitochondrial labeling and co-culture assays, we obtained evidence consistent with the release of astrocytic mitochondria, their uptake by injured neurons, and their association with the neuronal mitochondrial network. Building on this observation, we investigated whether direct delivery of functional astrocytic mitochondria could augment endogenous support during neuronal injury. Infused astrocytic mitochondria reduced oxidative stress, restored mitochondrial membrane potential, improved mitochondrial respiration, normalized calcium homeostasis, reduced early neuronal apoptosis, and restored mitochondrial mass. Astrocytic mitochondrial delivery enhanced dendritic arborization in primary neurons. *In vivo*, stereotactic infusion restored TH immunoreactivity in the SNpc and improved motor and non-motor behavioral deficits. These findings support the potential of astrocyte-derived mitochondria as a promising cell-free therapeutic approach for Parkinson’s disease.

## Highlights

• Astrocytes transfer mitochondria to injured neurons
• Astrocytic mitochondria restore neuronal bioenergetic function
• Mitochondrial delivery reduces oxidative stress and neuronal apoptosis
• Mitochondrial infusion improves neuronal morphology and behavior in PD rats

## Graphical abstract

**Figure:**
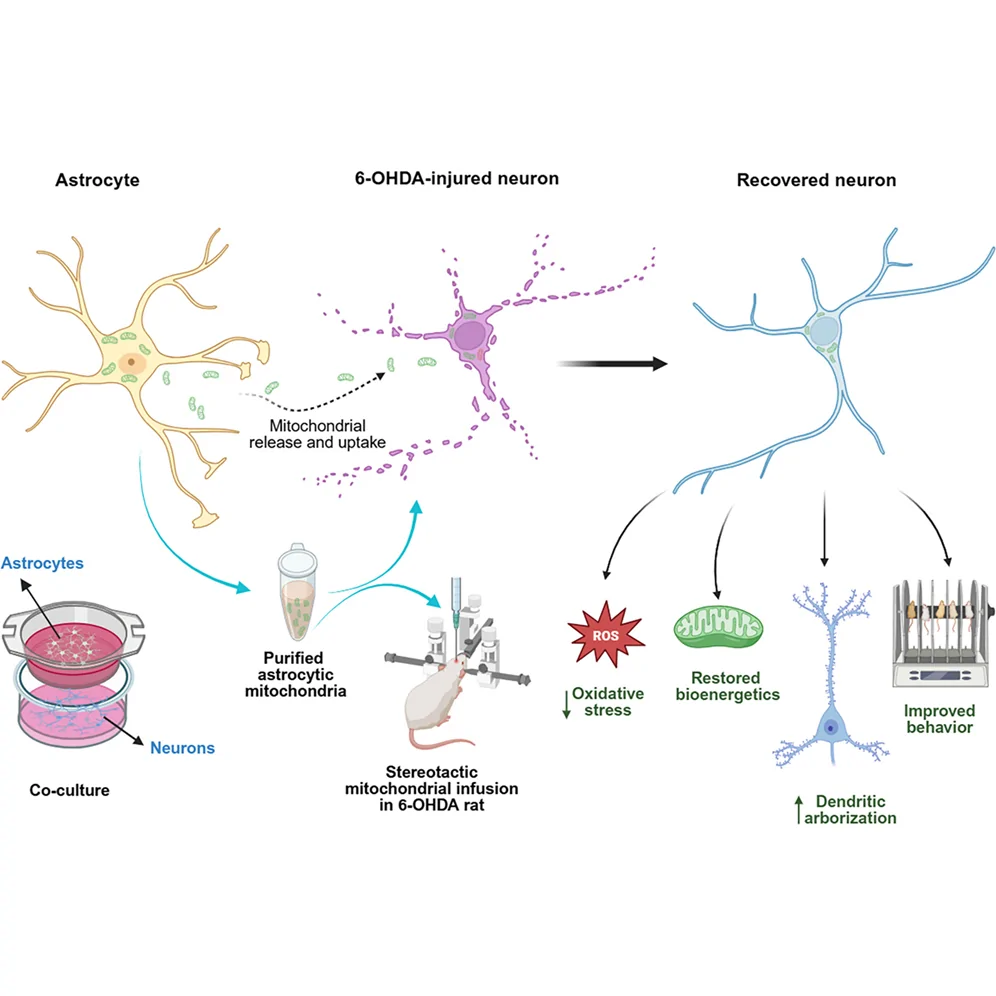

## Teaser

Neuroscience; Cell biology

## Introduction

Astrocytes are central nervous system (CNS) supporting and nurturing cells that play a role in neurodevelopment, neurotransmission, cerebral metabolism, blood flow, and neurogenesis.^1^ Astrocytes are the most common glial cells found throughout the brain, giving uniform, non-overlapping coverage in a network of astrocytes connected by gap junctions.^2^ Astrocytes are important for synaptic health because they drive synaptic development and positional accuracy,^3^^,^^4^ regulate transmission efficiency at pre- and post-synaptic locations, and modulate synapse formation and recycling.^5^^,^^6^^,^^7^^,^^8^ Until recently, astrocytes, along with other glial lineage cells such as oligodendrocytes and microglia, were thought to be structural cells whose primary job was to hold neurons together. Nonetheless, it is now known that astrocytes perform a variety of housekeeping roles, including the preservation of the extracellular environment and the stabilization of cell-cell communications in the CNS and tumor microenvironment.^9^^,^^10^ Understanding these functions has allowed an emphasis on the involvement of astrocytes in neurodegenerative disorders, particularly Parkinson’s disease (PD), which has led to astrocyte-specific studies with drug development potential.

Mitochondrial dysfunction is a major contributor to neurodegenerative diseases because mitochondria are essential for cellular energy production, calcium homeostasis, and neuronal survival.^11^ Therefore, a large number of the neuroprotective effects linked to astrocyte activation may include mitochondria. The development of defensive glial scars, the release of growth factors, and improved synaptic support are all believed to be critically reliant on the energy output of astrocytic mitochondria. Current findings have shown that if mitochondrial activity is disrupted, astrocytes cannot develop a protective proliferative response following acute brain injuries.^12^ Astrocytes, similar to most other cell types, eliminate their own damaged mitochondria through autophagy, also known as mitophagy, which is elevated during acute injury.^13^ This process may be employed to mitigate the deleterious consequences of oxidative stress and Ca^2+^ dysregulation, as previously mentioned. Astrocytes have the ability to take up and eliminate mitochondria released by other cells in addition to actively destroying their own damaged mitochondria. Davis et al. published the first report on this type of “transmitophagy.” They found that astrocytic lysosomes included a significant amount of fluorescently labeled mitochondria from retinal ganglion cells that were otherwise in a healthy state.^14^ Whether astrocytes are important for the elimination of neuron-derived mitochondria in neurodegenerative diseases or following traumatic brain injury is in question.

Mitochondrial intercellular transport appears to function both ways in astrocyte-neuron networks. Nevertheless, it seems that activated astrocytes can donate an amount of their functional mitochondria to neighboring damaged neurons, increasing neuronal survival, despite the fact that normally damaged mitochondria are passed from neurons to astrocytes for destruction. Astrocytes have been shown to release functional mitochondria into the extracellular environment through a calcium-dependent mechanism involving the CD38-cyclic ADP-ribose (cADPR) signaling pathway. These extracellular mitochondria can be taken up by neighboring neurons, particularly under conditions of ischemic injury, where they contribute to improved neuronal bioenergetics, survival, and recovery. Furthermore, experimental evidence suggests that mitochondrial protein O-GlcNAcylation contributes to maintaining the functional integrity and neuroprotective properties of astrocyte-derived extracellular mitochondria, highlighting astrocytes as a physiologically relevant source of reparative mitochondria in the injured CNS.^15^^,^^16^ Healthy cells, on the other hand, decay their own mitochondria; nonetheless, it has been discovered that retinal neurons send their mitochondria to astrocytes for disposal and recycling. Apart from the brain, mitochondrial mobilization has been documented in a few other circumstances. For example, bone marrow-derived stromal cells move mitochondria into pulmonary alveoli to decrease acute lung damage by restoring alveolar bioenergetics.^17^ As a result, it is essential to understand the role of liberated mitochondria in neurodegenerative diseases. We therefore hypothesized that astrocytes may transfer their mitochondria to neurons in a 6-hydroxydopamine (6-OHDA)-induced model of PD.

Numerous exogenous mitochondrial delivery methods, which include implanting healthy mitochondria into diseased or damaged tissue from an allogeneic or, preferably, autologous source, have been influenced by endogenous transcellular mitochondrial donation. Mitochondrial transplantation has been explored as a therapeutic approach for ischemia-reperfusion injury in pediatric patients with congenital heart disease,^18^ and it is currently the subject of intense clinical research. Although there are currently no relevant clinical trials of mitochondrial transplantation for CNS diseases, mitochondrial transplantation has shown notable functional and/or neuroprotective benefits in several animal models.^19^^,^^20^^,^^21^ Isolated mitochondria directly injected into glioma of live mice caused a metabolic transition from glycolysis to aerobic respiration along with a reduction in glioma development.^22^ More neuronal protection in the substantia nigra and improved locomotor functional performance were obtained in Parkinson’s model rats by using an improved transplant approach in conjunction with the peptide carrier protein PEP1 and mitochondrial delivery to the medial forebrain bundle.^21^ Healthy or functional mitochondria can be transplanted into injured rat spinal cords to support acute bioenergetics, but no long-term benefits for maintaining tissue or the recovery of vital sensory-motor functions have been found.^19^

Importantly, recent studies have also demonstrated that mitochondrial transplantation or mitochondrial transfer-based therapies confer neuroprotection in experimental models of PD, further supporting the concept that restoration of mitochondrial function represents a viable therapeutic strategy.^23^^,^^24^^,^^25^ While these studies establish the therapeutic feasibility of mitochondrial delivery, they primarily rely on exogenously supplied mitochondria and do not address whether endogenous glial cells serve as a physiologically relevant source of transferable mitochondria in PD pathology.

Building on these insights, we investigated whether exogenously delivered astrocyte-derived mitochondria could exert therapeutic effects in neurons compromised by neurotoxicity relevant to PD. Using *in vitro* and *in vivo* 6-OHDA models, we evaluated the capacity of astrocytic mitochondria to restore neuronal mitochondrial function, reduce oxidative and apoptotic stress, enhance neuronal structural integrity, and improve behavioral outcomes.

## Results

### Astrocyte-derived mitochondria attenuate oxidative stress and restore membrane potential in neurons

Oxidative stress is a key pathological feature in PD, contributing to mitochondrial dysfunction and dopaminergic neuronal death.^26^ Therefore, assessing reactive oxygen species (ROS) levels provides an important readout of cellular stress and mitochondrial health in neurodegenerative models. We examined whether astrocyte-derived mitochondria could protect neurons from oxidative stress induced by 6-OHDA, a commonly used neurotoxin model of PD. Using a transwell co-culture system (described in STAR Methods), primary cortical neurons at day 13 *in vitro* (DIV13) were treated with 40 μM 6-OHDA in the lower chamber, while astrocytes were seeded in the upper inserts. After 24 h of co-culture, ROS were measured using 2′,7′-dichlorodihydrofluorescein diacetate (DCFDA). Neurons in the presence of astrocytes showed a significant reduction in ROS levels compared to 6-OHDA-treated neurons cultured alone (Figures S1A and S1B). To further dissect the mechanism underlying this protection, we evaluated whether soluble factors/metabolites released by astrocytes contributed to the observed protective effect. When astrocyte-conditioned medium (ACM) was administered to 6-OHDA-treated neurons, a significant reduction in ROS levels was observed (Figures S1C and S1D), indicating the involvement of astrocyte-secreted components. However, the nature of these components remained unclear.

We next considered whether organelles like mitochondria within the ACM might play a role. ACM was processed by centrifugation and filtration through a 0.2 μm membrane to deplete mitochondrial content, generating mitochondrial-depleted ACM (md-ACM). The treatment of neurons with md-ACM failed to reduce ROS levels (Figures S1E and S1F), suggesting that mitochondria or mitochondria-like particulate components retained or disrupted during the depletion procedure, rather than small soluble factors, contribute substantially to the observed neuroprotective effects. To further assess the contribution of donor mitochondrial function, astrocytes were pretreated with mitochondrial division inhibitor-1 (mdivi-1, 50 μM for 12 h) prior to initiating co-culture. In this condition, astrocytes no longer attenuated ROS levels in 6-OHDA-exposed neurons, further supporting the requirement of active and functional mitochondrial signaling.

To determine whether direct mitochondrial infusion could restore protection, we isolated intact mitochondria from healthy astrocytes (as described in STAR Methods) and infused them into 6-OHDA-treated neurons that were co-cultured with mdivi-1-pretreated astrocytes. Remarkably, mitochondrial supplementation reversed the lack of protection caused by mdivi-1 and significantly reduced ROS levels (Figures 1A and 1B). These findings support an important contribution of astrocyte-derived mitochondria to buffering oxidative stress and promoting neuronal resilience under neurotoxic conditions.

**Figure 1:**
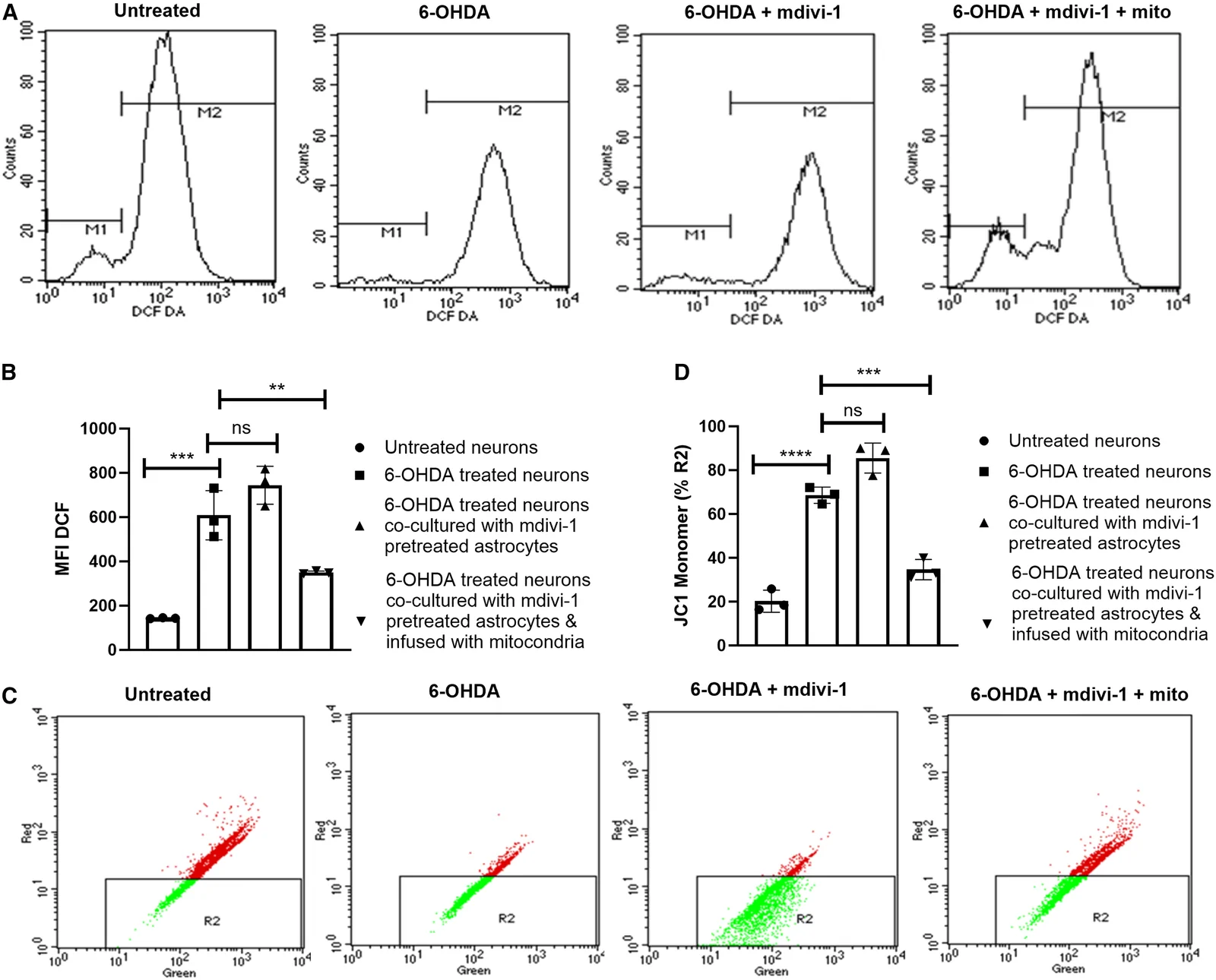
Astrocyte-derived mitochondria attenuate oxidative stress and restore mitochondrial membrane potential in neurons (A and B) Flow-cytometric analysis of intracellular reactive oxygen species (ROS) levels measured using DCF-DA. Representative histograms show mean fluorescence intensity (MFI) on the *x* axis and cell count on the *y* axis. Representative histograms are shown for the following conditions: untreated neurons, neurons treated with 6-OHDA, 6-OHDA-treated neurons co-cultured with mdivi-1-pretreated astrocytes and 6-OHDA-treated neurons co-cultured with mdivi-1-pretreated astrocytes following direct infusion of isolated astrocytic mitochondria. Bar graph (B) Quantitative analysis of DCF-DA fluorescence. (C) FACS-based single-cell analysis of mitochondrial membrane potential using the JC-1 dye presented as dot plots (green vs. red fluorescence). The R2 gate (green) represents cells with depolarized mitochondria characterized by increased JC-1 monomer fluorescence, whereas the red population corresponds to polarized, healthy mitochondria. Representative dot plots are shown for untreated neurons, 6-OHDA-treated neurons, 6-OHDA-treated neurons co-cultured with mdivi-1-pretreated astrocytes, and 6-OHDA-treated neurons co-cultured with mdivi-1-pretreated astrocytes following infusion of isolated astrocytic mitochondria. A minimum of 10,000 events was acquired per sample, and population shifts were analyzed as percentages of gated cells. (D) Quantitative analysis showing the percentage of cells within the R2 (depolarized) gate. Data are presented as mean ± SEM from three independent experiments (*n* = 3). Statistical analysis was performed using one-way ANOVA followed by Bonferroni post hoc test. Statistical significance is indicated as ⁎*p* < 0.05, ⁎⁎*p* < 0.01, ⁎⁎⁎*p* < 0.001, and ⁎⁎⁎⁎*p* < 0.0001; ns, not significant.

Mitochondrial membrane potential (MMP) serves as a key indicator of mitochondrial function and neuronal health. To assess changes in MMP, we used the mitochondrial membrane potential-sensitive fluorescent dye JC-1 (5,5′,6,6′-tetrachloro-1,1′,3,3′-tetraethylbenzimidazolylcarbocyanine iodide) and performed flow cytometric (FACS) analysis. Neurons treated with 6-OHDA and co-cultured with astrocytes displayed a significant restoration of MMP compared to 6-OHDA-treated neurons cultured without astrocytes (Figures S2A and S2B). Building on this, we examined whether astrocyte-derived mitochondria could reproduce this effect. Application of astrocyte conditioned medium (ACM) to 6-OHDA-treated neurons led to a significant increase in MMP (Figures S2C and S2D). In contrast, neurons treated with md-ACM failed to exhibit MMP recovery (Figures S2E and S2F), further supporting the contribution of mitochondria or mitochondria-like particulate components in ACM to this protective response.

Consistent with these findings, astrocytes pretreated with mdivi-1 were unable to support neuronal MMP restoration. However, direct infusion of isolated astrocytic mitochondria into 6-OHDA-treated neurons co-cultured with mdivi-1-pretreated astrocytes effectively rescued MMP levels (Figures 1C and 1D). These results further support a functional contribution of astrocytic mitochondria to maintaining mitochondrial integrity and promoting neuronal survival under neurotoxic stress.

### Astrocytic mitochondria reduce early-phase neuronal apoptosis under neurotoxic stress

Early neuronal apoptosis contributes to neuronal injury and neurotoxicity; therefore, we examined early apoptotic changes to determine whether astrocyte-derived mitochondria could protect neurons from 6-OHDA-induced damage.^27^ To investigate this, we measured early apoptotic changes using Annexin V-fluorescein isothiocyanate/propidium iodide (FITC/PI) staining by flow cytometry. Neurons exposed to 6-OHDA showed a marked increase in the early apoptotic cell population (lower right quadrant) compared with untreated neurons. A similar increase was observed when neuronal cultures were exposed to conditions in which astrocytic mitochondrial support was functionally compromised, including treatment with md-ACM or co-culture with mdivi-1-pretreated astrocytes.

Importantly, direct infusion of isolated astrocytic mitochondria into 6-OHDA-treated neurons significantly reduced the early apoptotic population under both mitochondrial-depleted and donor mitochondrial-impaired conditions (Figures 2A and 2B). These findings show that supplementation with isolated astrocytic mitochondria can attenuate early apoptotic injury in stressed neurons, even when endogenous astrocyte-derived mitochondrial support is disrupted. Together, the Annexin V-FITC/PI data support a protective role for astrocyte-derived mitochondria in preserving neuronal viability during early neurotoxic stress and provide functional evidence that mitochondrial supplementation can attenuate apoptosis-associated neuronal damage.

**Figure 2:**
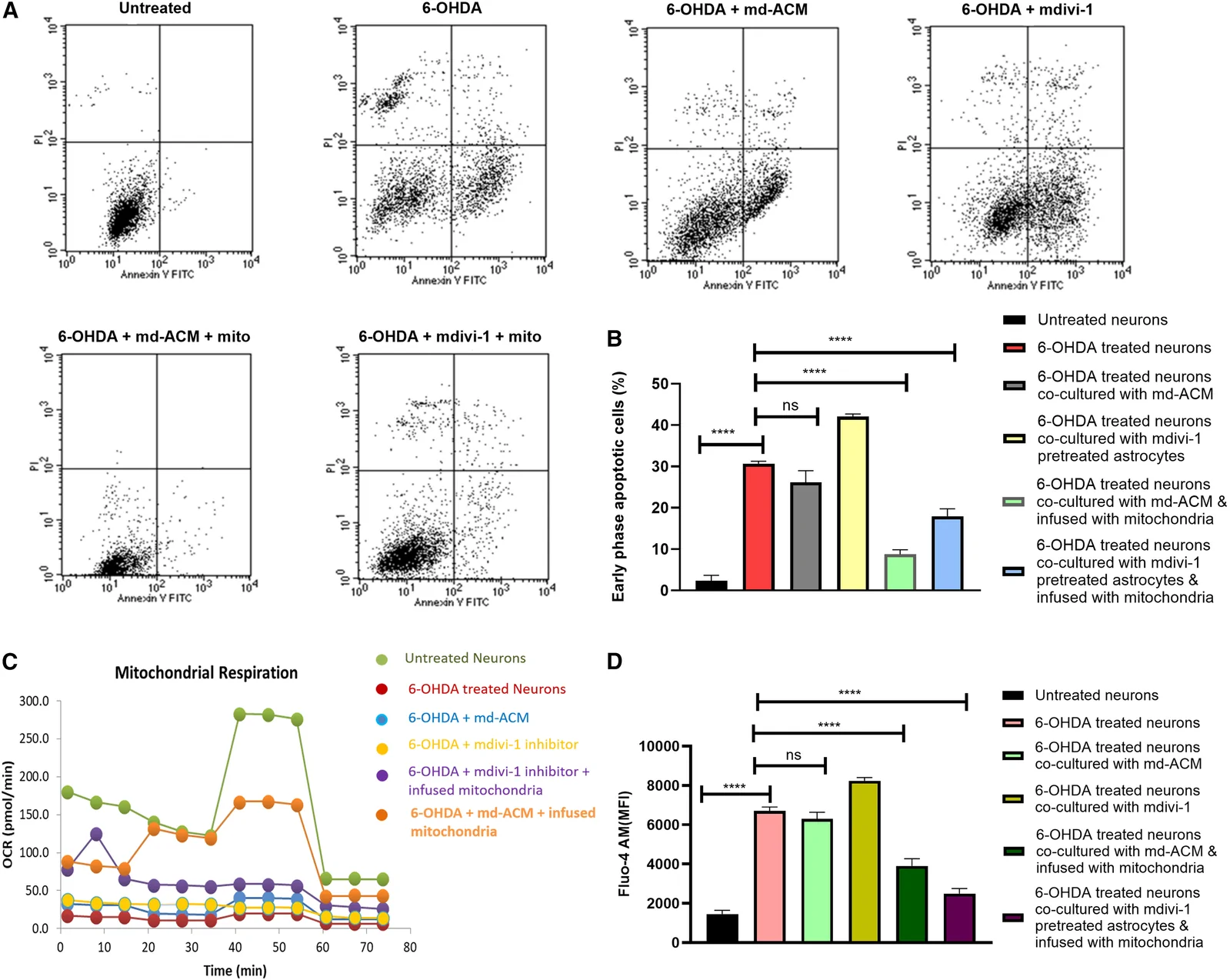
Astrocytic mitochondria reduce early-phase neuronal apoptosis and restore mitochondrial bioenergetics and calcium homeostasis (A) FACS analysis of early-phase neuronal apoptosis using Annexin V-FITC/PI. Representative plots are shown for the following conditions: untreated neurons, neurons treated with 6-OHDA, 6-OHDA-treated neurons treated with md-ACM, 6-OHDA-treated neurons co-cultured with mdivi-1-pretreated astrocytes, 6-OHDA-treated neurons co-cultured with md-ACM and infused with mitochondria, and 6-OHDA-treated neurons co-cultured with mdivi-1-pretreated astrocytes and infused with mitochondria. (B) Quantitative analysis of early apoptotic cells in the lower right quadrant. (C) Oxygen consumption rate (OCR) measured using an Agilent Seahorse XFp Analyzer. OCR traces represent untreated neurons (green line), 6-OHDA-treated neurons (red line), 6-OHDA-treated neurons exposed to md-ACM (blue line), 6-OHDA-treated neurons treated with mdivi-1 (yellow line), 6-OHDA-treated neurons exposed to md-ACM followed by infusion of isolated astrocytic mitochondria (purple line), and 6-OHDA-treated neurons treated with mdivi-1 followed by infusion of isolated astrocytic mitochondria (metallic gold line). (D) FACS analysis of intracellular calcium levels measured by Fluo-4 AM fluorescence and expressed as mean fluorescence intensity (MFI). Data are presented as mean ± SEM from three independent experiments (*n* = 3). Statistical analysis was performed using one-way ANOVA followed by Bonferroni post hoc test. Statistical significance is indicated as ⁎*p* < 0.05, ⁎⁎*p* < 0.01, ⁎⁎⁎*p* < 0.001, and ⁎⁎⁎⁎*p* < 0.0001; ns, not significant.

### Astrocytic mitochondria restore neuronal bioenergetics and calcium homeostasis

Neurons are highly energy-dependent and rely on mitochondria to generate ATP primarily through oxidative phosphorylation. The oxygen consumption rate (OCR) provides a measure of mitochondrial respiratory activity. To evaluate the effect of astrocytic mitochondrial supplementation on neuronal respiration, OCR was measured using the Agilent Seahorse XFp Analyzer. Because the Seahorse XFp assay was performed directly in XFp cell-culture miniplates, transwell co-culture was not used for this experiment. 6-OHDA-treated neurons exhibited a marked reduction in OCR compared with untreated neurons. Reduced OCR was also observed in 6-OHDA-treated neurons exposed to md-ACM or directly treated with mdivi-1. Notably, infusion of isolated astrocytic mitochondria increased OCR in 6-OHDA-treated neurons exposed to md-ACM or mdivi-1, supporting the ability of astrocytic mitochondrial supplementation to improve neuronal respiratory function under these experimental conditions (Figure 2C).

Given the importance of calcium homeostasis in synaptic transmission and neuronal signaling, we next examined intracellular calcium levels using Fluo-4 AM, a calcium-sensitive fluorescent dye. 6-OHDA-treated neurons showed elevated intracellular calcium levels compared with untreated neurons. Elevated calcium levels were also observed under conditions of impaired astrocytic mitochondrial support. In contrast, supplementation with isolated astrocytic mitochondria reduced intracellular calcium levels, supporting a protective effect of astrocytic mitochondrial supplementation on neuronal calcium homeostasis (Figure 2D).

We also assessed mitochondrial ROS as an indicator of cellular stress and early neuronal injury. Using MitoSOX Red, we observed a similar trend: infused astrocytic mitochondria substantially reduced mitochondrial ROS accumulation in 6-OHDA-treated neurons. Together, these findings show that astrocytic mitochondrial supplementation is associated with improved oxidative metabolism, stabilization of calcium homeostasis, and reduced mitochondrial oxidative stress under neurotoxic conditions (Figures S3A–S3D).

### Astrocytic mitochondria restore neuronal mitochondrial mass

Understanding how astrocytic mitochondria protect neurons requires investigating whether these organelles are directly transferred to neurons. We hypothesized that an increase in neuronal mitochondrial mass would reflect such a transfer. MitoTracker Green FM was used to assess mitochondrial mass/content in neurons under different treatment conditions. Exposure to 6-OHDA led to a marked decrease in mitochondrial mass compared to untreated neurons, indicating mitochondrial loss or damage. Interestingly, co-culturing 6-OHDA-treated neurons with astrocytes, or infusing them with isolated astrocytic mitochondria, resulted in a significant restoration of neuronal mitochondrial mass relative to 6-OHDA-treated neurons cultured alone (Figure S4).

These findings raised the question of whether mitochondrial infusion could restore mitochondrial mass even in the absence of support from functional astrocytes. When 6-OHDA-treated neurons were exposed to md-ACM or co-cultured with mdivi-1-pretreated astrocytes, a condition that normally fails to support mitochondrial recovery, mitochondrial mass remained low. However, infusion of astrocytic mitochondria under these conditions led to a significant increase in mitochondrial content (Figures 3A and 3B), supporting the idea that astrocytic mitochondrial transfer plays a key role in restoring neuronal mitochondrial integrity.

**Figure 3:**
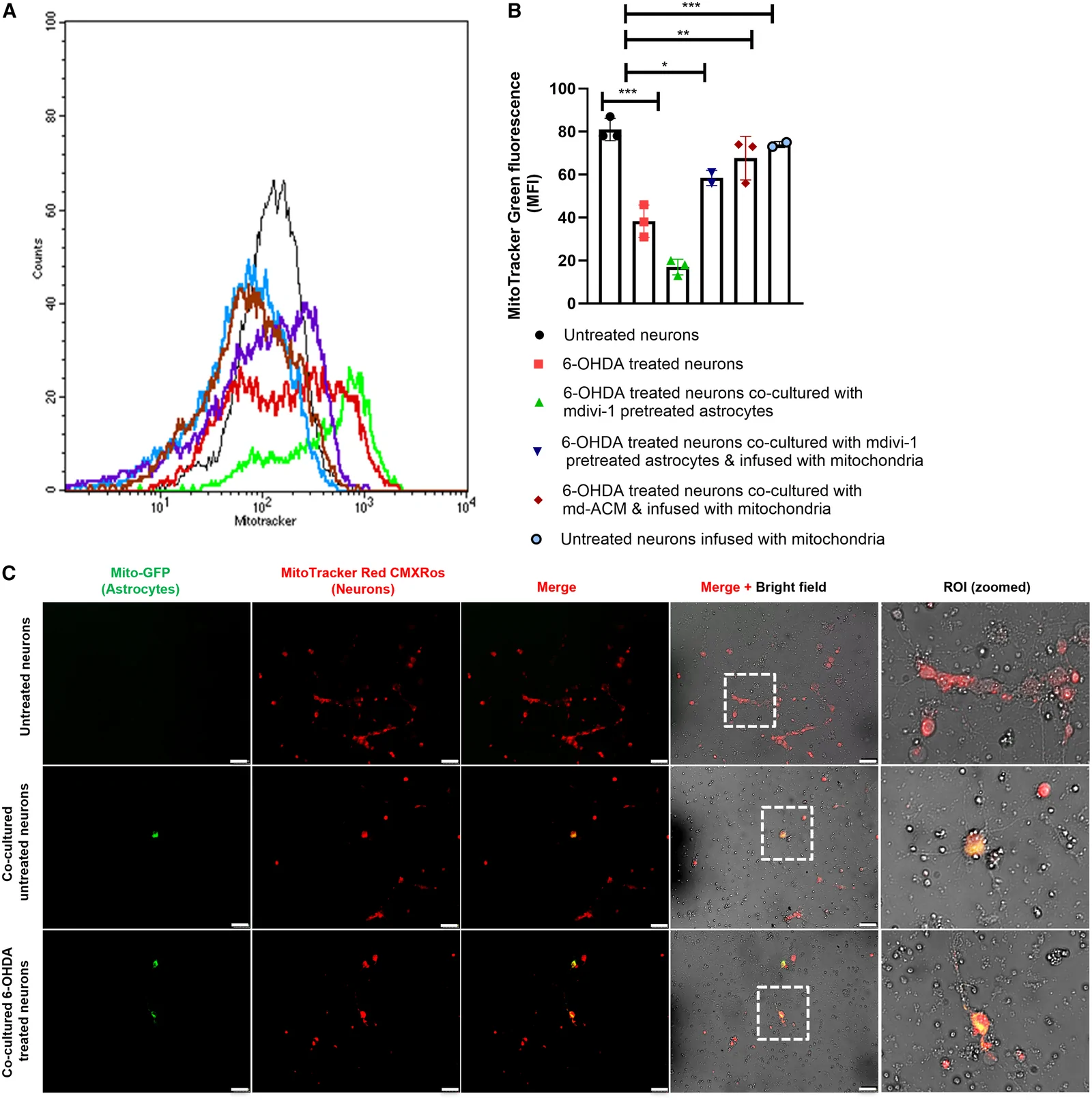
Evidence for astrocyte-derived mitochondria in recipient neurons (A) FACS analysis of mitochondrial mass measured as mean fluorescence intensity (MFI) using MitoTracker Green FM. Black line depicts untreated neurons, red line depicts neurons treated with 6-OHDA, green line depicts 6-OHDA-treated neurons co-cultured with mdivi-1-pretreated astrocytes, blue line depicts 6-OHDA-treated neurons co-cultured with mdivi-1-pretreated astrocytes and infused with mitochondria. Dark brown line depicts 6-OHDA-treated neurons treated with md-ACM and infused with mitochondria, and finally cyan blue line depicts untreated neurons infused with mitochondria. (B) Quantitative analysis of MitoTracker Green FM fluorescence. (C) Representative live-cell fluorescence images showing astrocyte-derived mitochondria labeled with mito-GFP (green) and neuronal mitochondria labeled with MitoTracker Red CMXRos (red) in untreated neurons, co-cultured untreated neurons, and co-cultured 6-OHDA-treated neurons. Merged images show co-localization of mito-GFP and MitoTracker Red CMXRos signals within the neuronal mitochondrial network. Merge + bright-field images show the cellular context, and white dashed boxes indicate regions of interest shown at higher magnification in the adjacent zoomed images. Images were captured using a Leica DMI 6000 microscope at 20× magnification. Scale bars, 50 μm. Data are presented as mean ± SEM from three independent experiments (*n* = 3). Statistical analysis was performed using one-way ANOVA followed by Bonferroni post hoc test. Statistical significance is indicated as ⁎*p* < 0.05, ⁎⁎*p* < 0.01, ⁎⁎⁎*p* < 0.001, and ⁎⁎⁎⁎*p* < 0.0001; ns, not significant.

### Evidence for astrocyte-derived mitochondria in recipient neurons

Building on our previous results showing that mitochondria present in ACM and directly infused astrocytic mitochondria confer protection to 6-OHDA-injured neurons, we further investigated the mechanism underlying this neuroprotection. The lack of protection observed in conditions involving md-ACM or mdivi-1-pretreated astrocytes suggested that intact astrocytic mitochondria may be responsible for the neuroprotection, potentially via direct transfer to neurons. Initial support for this hypothesis came from experiments using MitoTracker Green FM, which revealed a significant increase in mitochondrial mass in neurons co-cultured with astrocytes or infused with astrocytic mitochondria. To directly visualize mitochondrial transfer, astrocytes were pre-labeled with MitoTracker Red CMXRos, a fluorescent marker for functional mitochondria, and then co-cultured with either untreated or 6-OHDA-treated neurons for 24 h. Following extensive washing and live-cell imaging, MitoTracker Red CMXRos positive mitochondrial signals derived from labeled astrocytes were detected in association with recipient neurons. Importantly, the number of transferred mitochondria was significantly higher in 6-OHDA-treated neurons than in untreated controls (Figure S5).

We next examined whether the transferred astrocytic mitochondria were internalized by neurons or became closely associated with neuronal mitochondria. To check this, astrocytic mitochondria were labeled using a mito-GFP construct, which targets the mitochondrial matrix via the E1α pyruvate dehydrogenase leader sequence, delivered using BacMam 2.0 technology. Neurons, in parallel, were labeled with MitoTracker Red CMXRos. Upon co-culture, mito-GFP-labeled astrocytic mitochondria were found in close proximity to the neuronal soma and were more abundant in 6-OHDA-treated neurons compared to untreated neurons. Moreover, co-localization imaging revealed overlap between mito-GFP and MitoTracker Red signals, consistent with close association of astrocyte-derived mitochondria with the neuronal mitochondrial network (Figure 3C). However, these imaging data do not by themselves establish mitochondrial fusion.

FACS analysis was used to further validate the transfer of astrocytic mitochondria to neurons. Neuronal events double-positive for mito-GFP and MitoTracker Red CMXRos were detected and sorted, providing additional evidence consistent with neuronal uptake of astrocyte-derived mitochondrial material (Figures 4A–4C).

**Figure 4:**
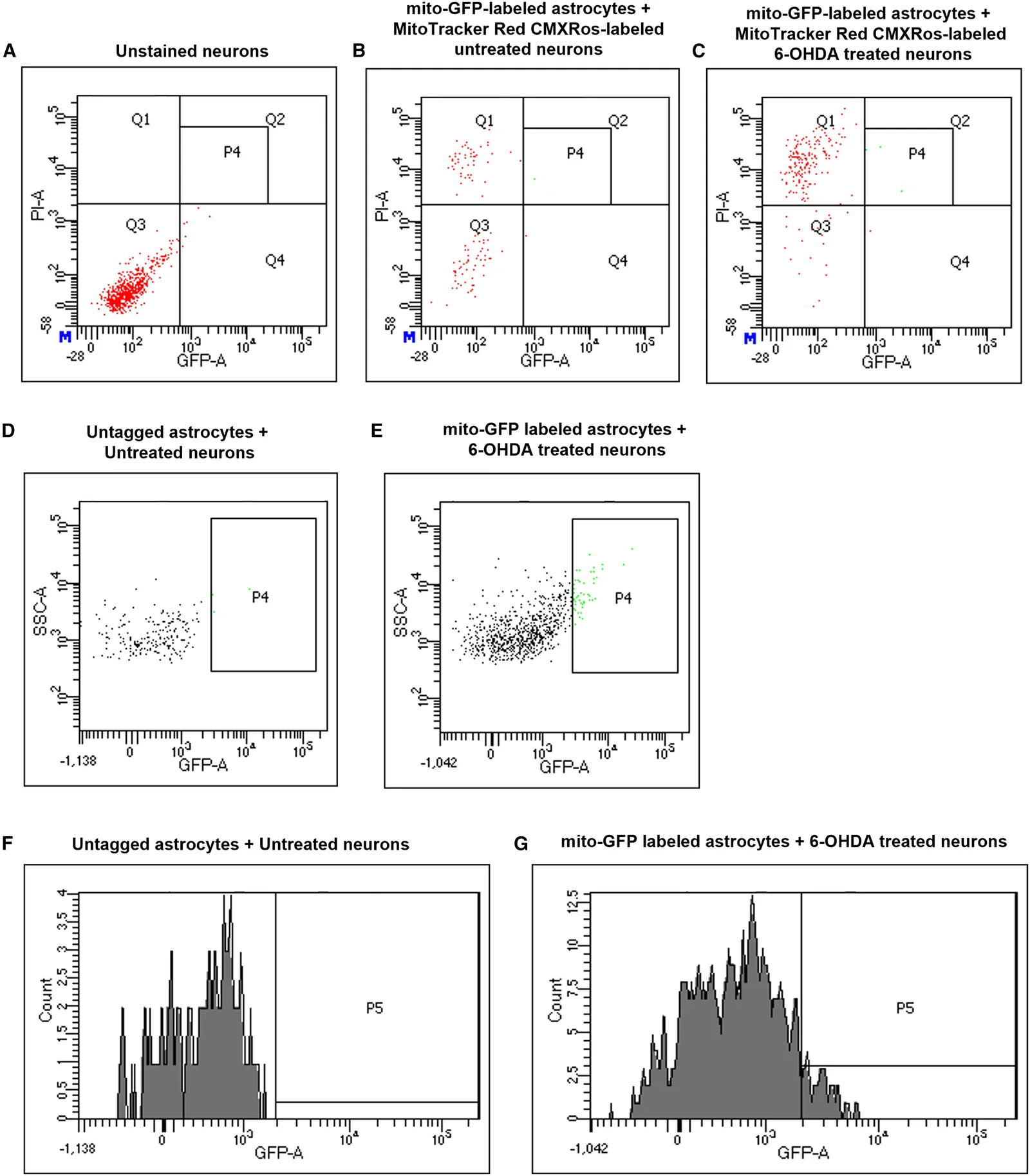
Flow cytometry-based detection and sorting of astrocyte-derived mito-GFP-labeled mitochondrial material in neurons and conditioned media (A) Representative flow cytometry plot of unstained neuronal cells used to define background fluorescence and gating parameters. (B) Representative dot plot showing neurons co-cultured with astrocytes, with Q2/P4 gating identifying double-positive events for mito-GFP (astrocytic mitochondria) and MitoTracker Red CMXRos (neuronal mitochondrial network). (C) Representative dot plot showing 6-OHDA-treated neurons co-cultured with astrocytes, with Q2/P4 gating highlighting mito-GFP and MitoTracker Red CMXRos double-positive neuronal events, providing evidence for neuronal uptake of astrocyte-derived mitochondrial material. (D and E) Representative flow cytometry plots illustrating the detection and sorting of mito-GFP labeled extracellular mitochondrial particles in astrocyte-conditioned medium (ACM) collected from the indicated experimental conditions. P4 gating denotes a purified population of mito-GFP positive particles, while P5 shows separation of labeled mitochondrial particles from the total extracellular population. (F) Representative plot of conditioned medium collected from untreated astrocyte cultures. (G) Representative plot of mito-GFP-tagged astrocyte-conditioned medium collected following co-culture with 6-OHDA-treated neurons, showing enrichment of labeled extracellular mitochondrial particles. All images represent qualitative flow cytometry plots used to illustrate gating strategies and population distributions.

In addition to intracellular transfer, we investigated the presence of mitochondria in the extracellular space. The conditioned medium was collected from mito-GFP-labeled astrocytes co-cultured with 6-OHDA-treated neurons after 24 h and compared to ACM from untagged astrocytes co-cultured with untreated neurons. Nanometer-sized, mitochondria-like particles were detected in both conditions, with a significantly higher abundance observed in the ACM from 6-OHDA-treated co-cultures (Figures 4D–4G).

Together, these findings provide evidence consistent with the release of astrocyte-derived mitochondrial material into the extracellular space and its increased association with recipient neurons under neurotoxic stress. The observed intracellular labeling and co-localization with the neuronal mitochondrial network are compatible with mitochondrial uptake and possible integration, although the precise intracellular fate of the transferred material was not directly resolved.

### Astrocytic mitochondria enhance dendritic arborization and neuronal structural recovery

After obtaining evidence supporting the transfer of astrocytic mitochondria to neurons, we next investigated whether this mitochondrial support translated into structural improvement in 6-OHDA-treated neurons. Dendritic arborization is a key determinant of neuronal connectivity, synaptic integration, and functional recovery, and it is commonly disrupted under neurotoxic stress in PD-related models. Therefore, we assessed neuronal morphology using microtubule-associated protein 2 (MAP2) immunostaining followed by Sholl analysis.

6-OHDA exposure markedly reduced dendritic complexity, as reflected by decreased dendritic branching and fewer intersections from the neuronal soma. Similar impairment was observed under conditions where astrocytic mitochondrial support was compromised. Importantly, infusion of isolated astrocytic mitochondria significantly restored dendritic arborization, increasing both dendritic length and mean intersection count (Figures 5A–5C). These findings indicate that astrocyte-derived mitochondria not only improve neuronal survival but also promote structural recovery of injured neurons, supporting a potential role in preserving structural features associated with neuronal connectivity under neurotoxic stress.

**Figure 5:**
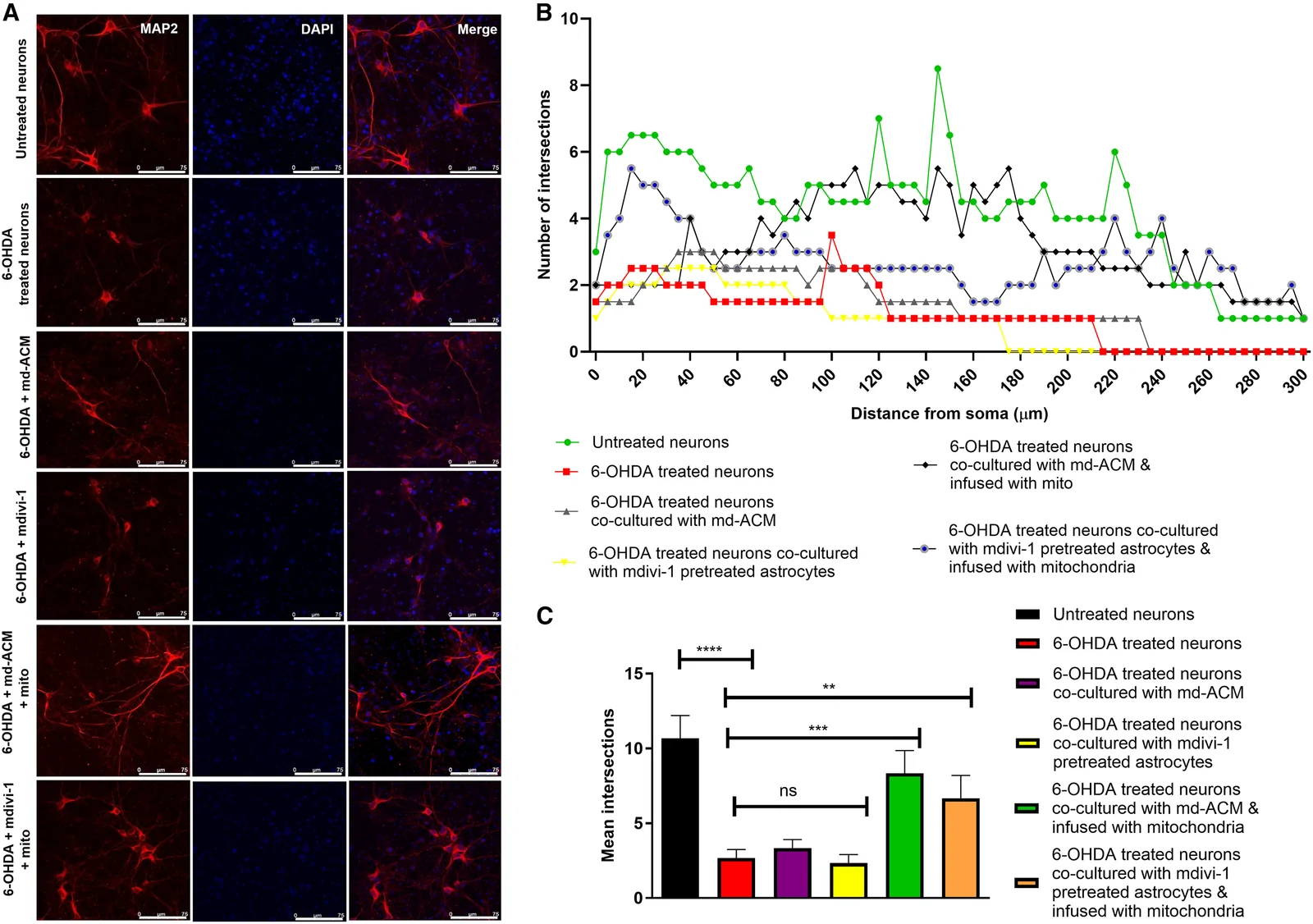
Astrocytic mitochondrial infusion increases dendritic arborization (A) Representative confocal microscopy images showing MAP2 expression (red) co-localized with the nuclear marker DAPI (blue) in primary neurons under different experimental conditions: untreated neurons, 6-OHDA-treated neurons, 6-OHDA-treated neurons co-cultured with md-ACM, 6-OHDA-treated neurons co-cultured with mdivi-1-pretreated astrocytes, 6-OHDA-treated neurons co-cultured with md-ACM and infused with astrocytic mitochondria, and 6-OHDA-treated neurons co-cultured with mdivi-1-pretreated astrocytes and infused with astrocytic mitochondria. Scale bars, 75 μm. (B) Sholl analysis showing dendritic arborization, represented as the number of dendritic intersections at increasing distances from the neuronal soma. (C) Representative bar graph showing quantitative analysis of mean dendritic intersections across experimental groups. Data are presented as mean ± SEM from three independent experiments (*n* = 3). Statistical analysis was performed using one-way ANOVA followed by Bonferroni post hoc test. Statistical significance is indicated as ⁎*p* < 0.05, ⁎⁎*p* < 0.01, ⁎⁎⁎*p* < 0.001, and ⁎⁎⁎⁎*p* < 0.0001; ns, not significant.

### Astrocytic mitochondria restore neuroprotective markers associated with PD pathogenesis

We further evaluated the protective effects of astrocytic mitochondria by assessing their impact on key markers associated with PD pathogenesis. This included MAP2, a marker of neuronal structural integrity; tyrosine hydroxylase (TH), the rate-limiting enzyme in catecholamine biosynthesis; and cytochrome *c* oxidase subunit 4 (COX4), a critical component of the mitochondrial electron transport chain that is known to be compromised following 6-OHDA exposure. Immunocytochemical analysis revealed a significant reduction in MAP2 and COX4 expression in neurons treated with 6-OHDA, as well as in those exposed to md-ACM or co-cultured with mdivi-1-pretreated astrocytes. COX4 mean fluorescence intensity (MFI) was quantified specifically within MAP2 positive neuronal regions of interest to ensure neuron-restricted analysis. Notably, infusion of isolated astrocytic mitochondria restored the expression of both MAP2 and COX4 in these neurons (Figures 6A and 6B), suggesting that mitochondrial transfer supports both cytoskeletal stability and mitochondrial respiratory function.

**Figure 6:**
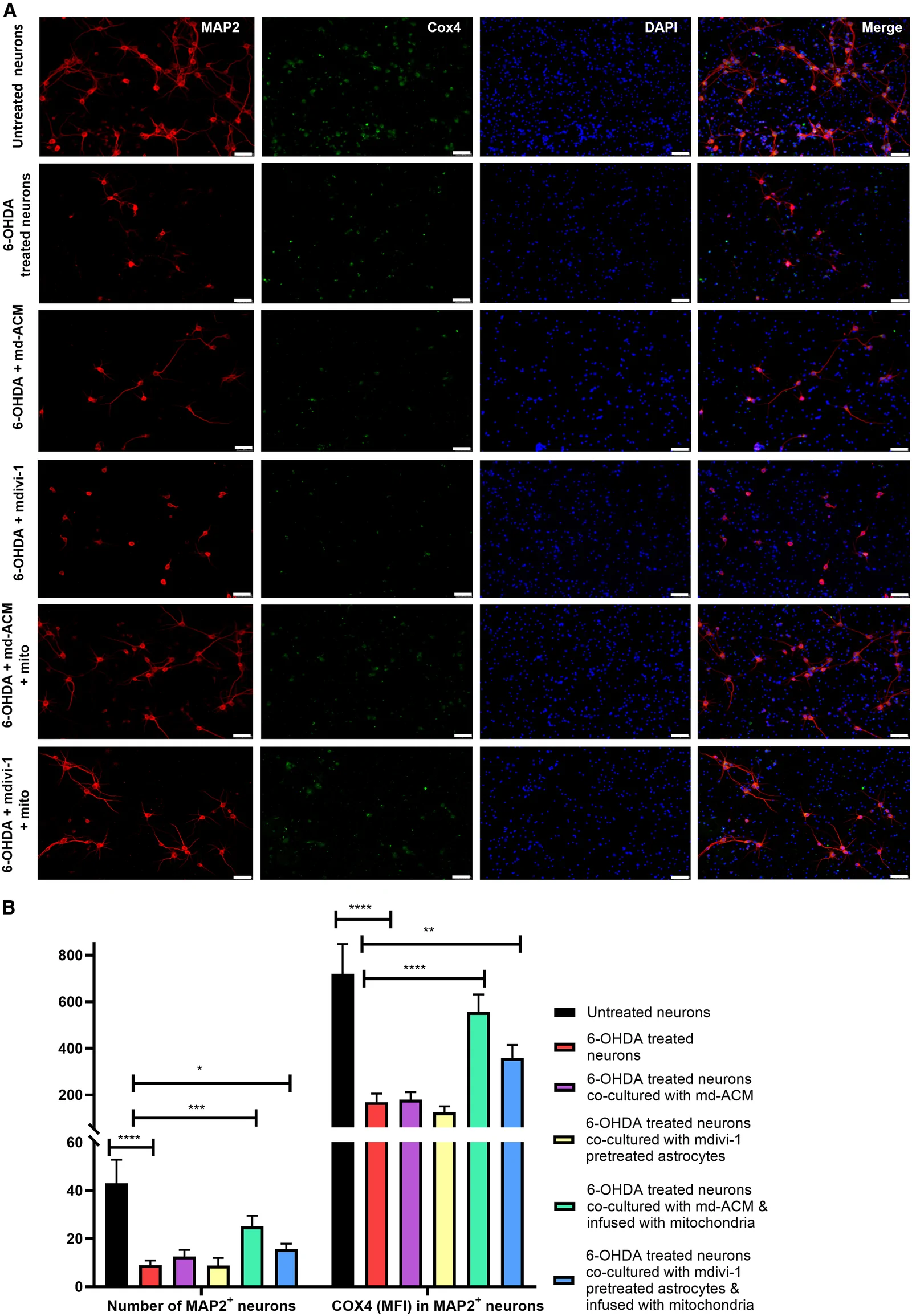
Astrocytic mitochondria restore neuroprotective markers associated with PD pathogenesis (A) Representative immunocytochemistry (ICC) images showing MAP2 and COX4 expression co-localized with the nuclear marker DAPI in primary neurons. Experimental groups include untreated neurons, 6-OHDA-treated neurons, 6-OHDA-treated neurons treated with md-ACM, 6-OHDA-treated neurons co-cultured with mdivi-1-pretreated astrocytes, 6-OHDA-treated neurons co-cultured with md-ACM and infused with astrocytic mitochondria, and 6-OHDA-treated neurons co-cultured with mdivi-1-pretreated astrocytes and infused with astrocytic mitochondria. Images were captured using a Leica DMI6000 microscope at 20× magnification. Scale bars, 50 μm. (B) Quantification of the number of MAP2-positive neurons and COX4 mean fluorescence intensity (MFI) within MAP2-positive neuronal regions. Data are presented as mean ± SEM from three independent experiments (*n* = 3). Statistical analysis was performed using one-way ANOVA / two-way ANOVA followed by Bonferroni post hoc test. Statistical significance is indicated as ⁎*p* < 0.05, ⁎⁎*p* < 0.01, ⁎⁎⁎*p* < 0.001, and ⁎⁎⁎⁎*p* < 0.0001; ns, not significant.

To extend these *in vitro* findings to an *in vivo* context and assess dopaminergic integrity in the substantia nigra pars compacta (SNpc), we next evaluated the effect of astrocytic mitochondrial delivery on TH expression in this region, a hallmark pathological feature of PD. Mitochondria were isolated from primary astrocyte cultures derived from neonatal SD rat pups and labeled with MitoTracker Red CMXRos, which is suitable for labeling functional mitochondria in live cells. Labeled astrocytic mitochondria were stereotaxically infused into the SNpc at a dose of approximately 1 × 10^5^ mitochondria in 5 μL, administered via a cannula on days 0, 7, and 14. Given the limited intracellular retention and stability of MitoTracker Red CMXRos, mitochondrial labeling was performed 24 h prior to sacrifice to confirm regional localization within the SNpc.

Immunohistochemical analysis revealed that 6-OHDA-lesioned animals receiving astrocytic mitochondrial infusions exhibited a significant restoration of TH immunoreactivity in the SNpc compared with animals treated with 6-OHDA alone. 6-OHDA-lesioned rats receiving mitochondria isolated from mdivi-1-pretreated astrocytes showed reduced TH immunoreactivity, whereas subsequent delivery of functional astrocytic mitochondria significantly restored TH immunoreactivity, supporting a neuroprotective effect associated with functional astrocytic mitochondrial delivery (Figures 7A and 7B).

**Figure 7:**
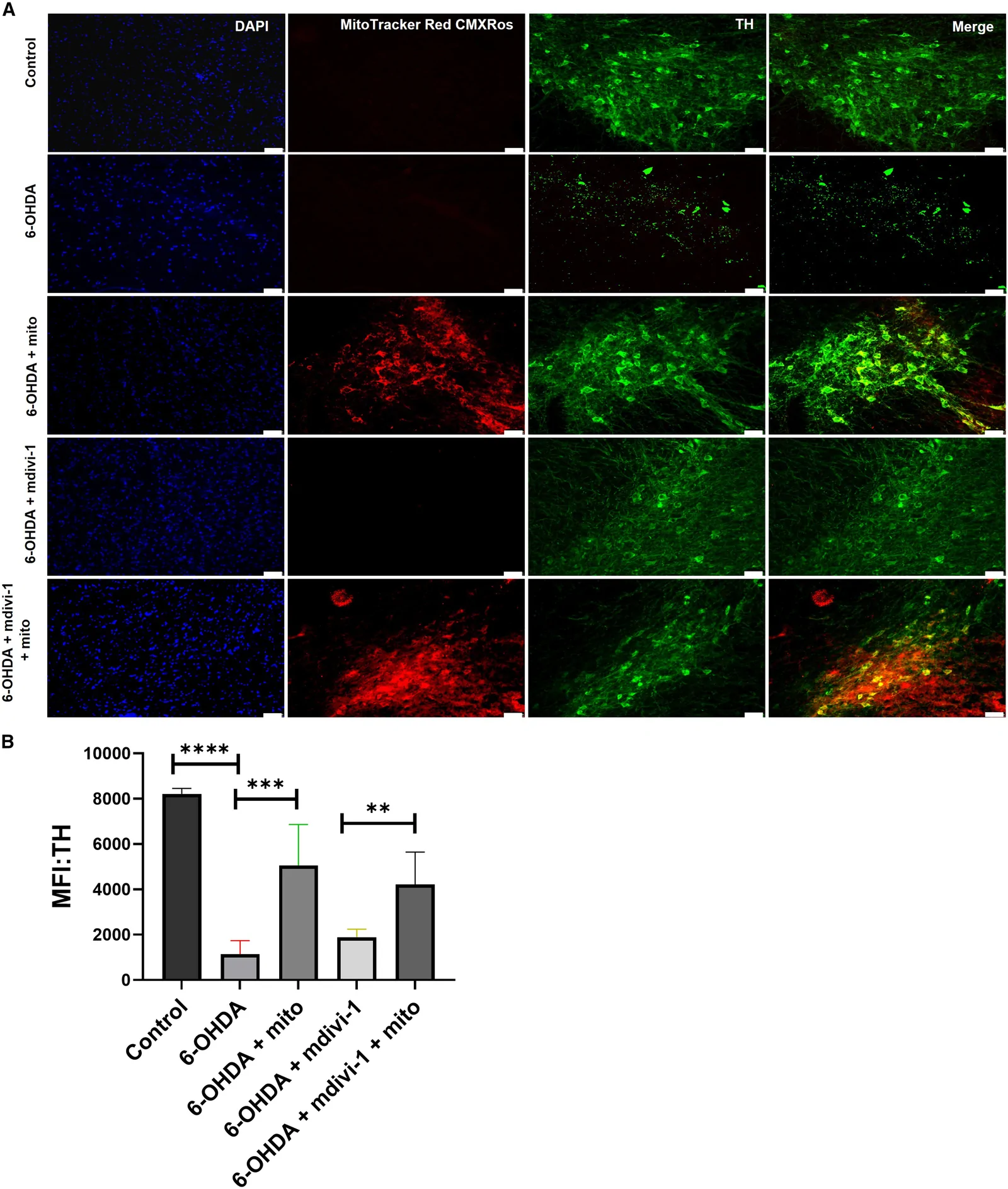
Astrocytic mitochondrial infusion restores tyrosine hydroxylase expression in the substantia nigra pars compacta (A) Representative immunohistochemical (IHC) images showing TH expression in the SNpc region of rat brain sections. Experimental groups include control animals, 6-OHDA-lesioned animals, 6-OHDA-lesioned animals receiving functional astrocytic mitochondria (6-OHDA + Mito), 6-OHDA-lesioned animals receiving mitochondria isolated from mdivi-1-pretreated astrocytes (6-OHDA + mdivi-1), and 6-OHDA-lesioned animals receiving both mdivi-1-pretreated astrocytic mitochondria and functional astrocytic mitochondria (6-OHDA + mdivi-1 + Mito). Images were captured using a Leica DMI6000 microscope at 20× magnification. Scale bars, 50 μm. (B) Quantitative analysis of TH immunoreactivity measured as mean fluorescence intensity (MFI) within TH-positive regions across experimental groups. Data are presented as mean ± SEM; *n* = 8 adult male Sprague-Dawley rats per group, with 3–5 SNpc sections analyzed per brain. Statistical analysis was performed using one-way ANOVA followed by Bonferroni post hoc test. Statistical significance is indicated as ⁎*p* < 0.05, ⁎⁎*p* < 0.01, ⁎⁎⁎*p* < 0.001, and ⁎⁎⁎⁎*p* < 0.0001; ns, not significant.

Together, these findings support a neuroprotective effect of astrocyte-derived mitochondrial delivery on PD-associated molecular deficits and dopaminergic neuronal integrity under the experimental neurotoxic conditions examined *in vitro* and *in vivo*.

### Astrocytic mitochondrial delivery improves motor and non-motor behavioral outcomes in 6-OHDA-lesioned rats

Motor and non-motor symptoms are hallmark features of PD, contributing significantly to disease burden and affecting patients’ quality of life. While motor symptoms such as bradykinesia and postural instability are well-characterized, non-motor symptoms including social withdrawal and anxiety are often under-recognized despite their clinical relevance. To comprehensively evaluate the therapeutic potential of astrocytic mitochondria, we assessed their effects on both motor and non-motor behavioral phenotypes in a 6-OHDA-induced rat model of PD. On day 28, open-field activity was conducted to assess spontaneous locomotor activity by measuring total distance traveled (xy-ambulatory) and rearing behavior (z total) over a 30-min session. Rats lesioned with 6-OHDA, as well as 6-OHDA-lesioned rats receiving mitochondria isolated from mdivi-1-pretreated astrocytes, exhibited a significant reduction in both parameters compared to control animals. However, delivery of astrocytic mitochondria into the SD rat brain in both groups markedly reversed these deficits, indicating improved locomotor function. Motor coordination was further assessed using the rotarod test. The latency to fall was significantly increased in rats receiving astrocytic mitochondria, suggesting enhanced neuromuscular coordination and balance (Figures 8A–8C).

**Figure 8:**
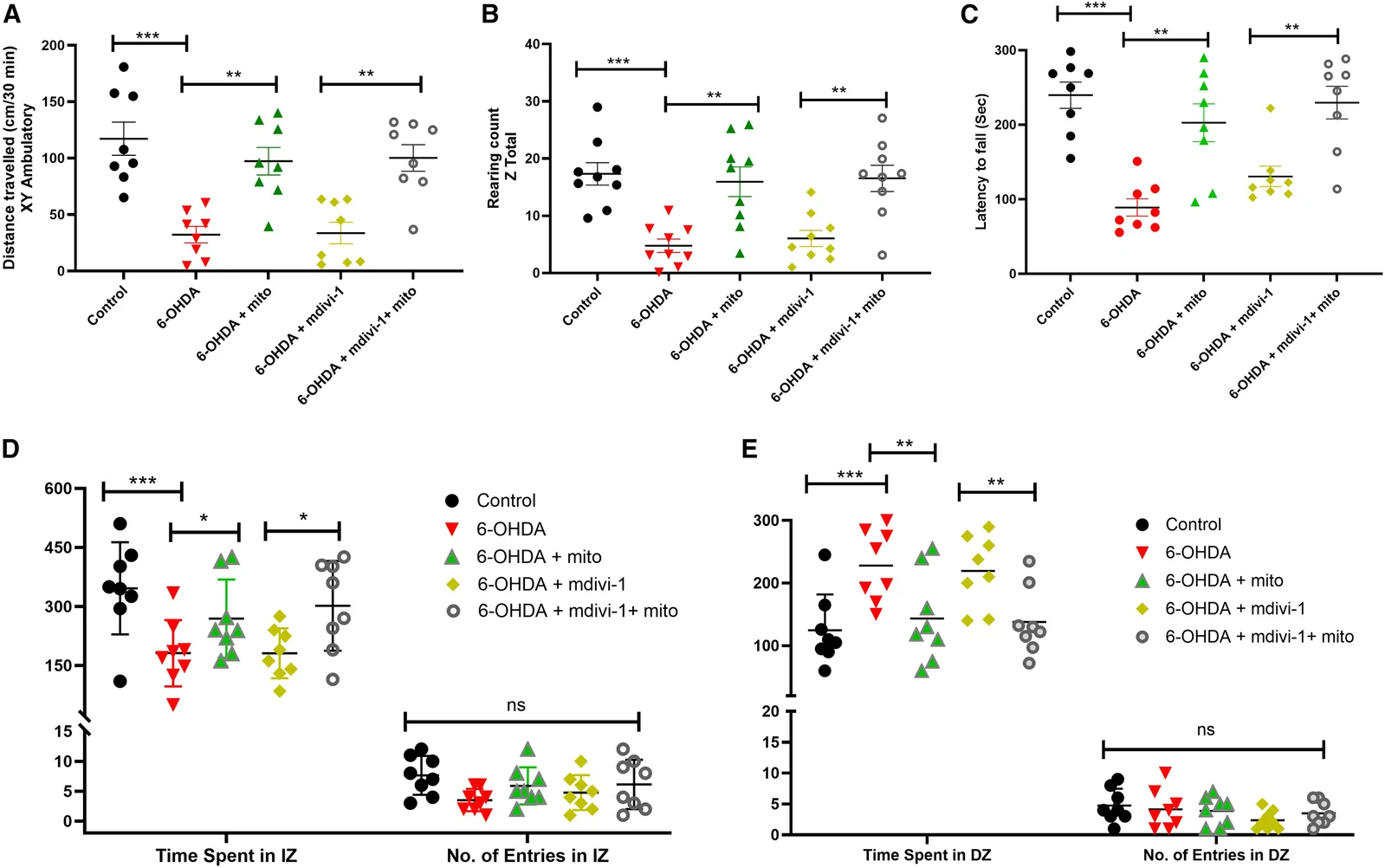
Astrocytic mitochondrial delivery improves motor and non-motor behavioral outcomes in 6-OHDA-lesioned rats Motor behavior was assessed using open-field activity and rotarod tests on day 28 after 6-OHDA surgery. (A) Distance traveled along the *x*/*y* axis over a 30 min period in the open-field arena. (B) Total rearing activity counts along the *z* axis over a 30 min period in the open-field arena. (C) Latency to fall (seconds) in the rotarod test. Non-motor behavioral symptoms were assessed using social interaction and light/dark box tests on day 27 after 6-OHDA surgery. (D) Total time spent and number of entries in the interaction zone (IZ). (E) Total time spent and number of entries in the dark zone. Data are presented as mean ± SEM; *n* = 8 adult male Sprague-Dawley rats per group. Statistical analysis was performed using one-way ANOVA followed by Bonferroni post hoc test. Statistical significance is indicated as ⁎*p* < 0.05, ⁎⁎*p* < 0.01, ⁎⁎⁎*p* < 0.001, and ⁎⁎⁎⁎*p* < 0.0001; ns, not significant.

On day 27, non-motor symptoms were assessed using the social interaction and light/dark box tests to evaluate social and anxiety-like behaviors. Rats lesioned with 6-OHDA, as well as 6-OHDA-lesioned rats receiving mitochondria isolated from mdivi-1-pretreated astrocytes, exhibited reduced sociability, indicated by decreased time spent in the interaction zone (IZ), and increased anxiety-like behavior, as reflected by greater time spent in the dark zone (DZ). Administration of functional astrocytic mitochondria in both groups significantly improved these behavioral outcomes, increasing the time spent in the IZ and reducing the time spent in DZ. Importantly, the number of entries into either zone did not differ significantly across groups, suggesting that the observed effects were specific to social and anxiety-like behavior rather than changes in overall locomotor activity (Figures 8D and 8E).

Together, these findings show that astrocytic mitochondrial delivery improves motor and non-motor behavioral outcomes in the 6-OHDA rat model, supporting further investigation of its therapeutic potential.

## Discussion

Astrocytes have traditionally been viewed as only supportive cells in the CNS, but an expanding body of evidence suggests they are far more dynamic, particularly in their ability to respond to neuronal injury and modulate recovery. In our study, we provide evidence consistent with astrocyte-to-neuron mitochondrial transfer in 6-OHDA-induced models of PD, together with robust neuroprotective effects of astrocytic mitochondrial delivery at cellular and behavioral levels.

Previous research has shown that astrocytes actively engage in mitochondrial quality control, not only removing their own damaged mitochondria via mitophagy^13^ but also participating in the degradation of neuron-derived mitochondria through a process known as *trans*-mitophagy.^14^ More recently, astrocytes have been reported to donate mitochondria to neurons following ischemic injury,^15^ suggesting a potential bidirectional trafficking system that may serve as a reparative mechanism. Our data build on this concept and extend it to the context of PD, providing evidence consistent with the transfer of astrocyte-derived mitochondrial material to damaged neurons and demonstrating neuroprotective effects associated with functional astrocytic mitochondrial delivery.

We used both mito-GFP and MitoTracker Red CMXRos labeling strategies to visualize this transfer. Co-localization of astrocytic and neuronal mitochondrial signals (Figures 3C and S5) was consistent with close association of astrocyte-derived mitochondria with the neuronal mitochondrial network, particularly under neurotoxic stress; however, these imaging data do not directly demonstrate mitochondrial fusion or stable integration. Notably, astrocyte-derived mitochondrial signals were detected in association with the neuronal soma, consistent with neuronal uptake; however, the precise intracellular fate of the transferred mitochondrial material remains to be established. Importantly, mitochondrial delivery was accompanied by significant improvements in neuronal mitochondrial function. Neurons receiving astrocytic mitochondria exhibited significantly reduced ROS (Figures 1A, 1B, and S1) and restored MMP levels (Figures 1C, 1D, and S2). These findings align with previous observations that astrocytes mitigate oxidative damage during acute brain injury.^12^^,^^26^

Although the present study provides evidence supporting astrocyte-to-neuron mitochondrial transfer and its functional neuroprotective significance, the precise molecular mechanisms governing mitochondrial release from astrocytes and uptake by neurons were not directly interrogated. The experimental design was focused on obtaining evidence supporting mitochondrial transfer and evaluating its functional consequences under neuronal injury, rather than dissecting the underlying pathways mediating these processes. Importantly, all co-culture experiments were performed using a transwell system that physically separates astrocytes and neurons, thereby excluding direct cell-contact-dependent mechanisms such as tunneling nanotubes, while permitting passage of extracellular particles or vesicles below the transwell pore-size limit. Instead, our data support a model in which astrocytes release mitochondria or mitochondria-containing particles into the extracellular space that may subsequently associate with or be taken up by neurons through contact-independent mechanisms. Potential uptake pathways, including endocytosis or macropinocytosis, remain to be investigated. This interpretation is supported by the detection of mito-GFP positive extracellular mitochondrial particles in ACM, the loss of neuroprotection upon depletion of particulate organelles in mitochondrial-depleted conditioned medium, and the restoration of neuronal mitochondrial function following direct mitochondrial add-back. Accordingly, while potential mechanisms of mitochondrial release and neuronal uptake are discussed, these pathways were not experimentally dissected in the present study and represent important directions for future investigation. While future studies will be required to define the molecular machinery governing mitochondrial release and uptake in greater detail, our findings support a functional contribution of astrocyte-derived mitochondrial transfer and its potential therapeutic relevance in astrocyte-neuron communication under conditions of neuronal injury.

Importantly, md-ACM was employed as a functional exclusion control rather than as a selective mitochondrial purification strategy. This approach was designed to distinguish the contribution of soluble astrocyte-derived factors from particulate, mitochondria-like structures. The failure of md-ACM to confer neuroprotection, together with robust rescue observed following mitochondrial add-back, indicates that soluble factors alone are insufficient to explain the observed neuronal recovery. Moreover, mitochondrial fission itself is not proposed as the regenerative mechanism in this study. Instead, mdivi-1 was used as a tool to functionally perturb astrocytic mitochondria and assess mitochondrial dependence of the observed neuroprotective effects. Nevertheless, the non-selective nature of the md-ACM depletion procedure and the pharmacological limitations of mdivi-1 should be considered when interpreting these experiments.

A notable observation in our study was the capacity of astrocytic mitochondria to restore intracellular calcium balance. Calcium dysregulation is a well-known hallmark of excitotoxicity in neurodegenerative diseases. Following infusion of astrocytic mitochondria, we observed a significant reduction in calcium overload in 6-OHDA-treated neurons (Figure 2D), suggesting that these organelles contribute to homeostatic calcium buffering. This is consistent with the role of mitochondrial Ca^2+^ uptake in astrocyte function, particularly in vascular remodeling and neuroprotection.^28^

Moreover, infusion of astrocyte-derived mitochondria significantly reduced early-stage neuronal apoptosis, as demonstrated by Annexin V-FITC flow cytometry. These findings suggest that astrocytic mitochondria help rescue neurons and promote their survival under 6-OHDA-induced neurotoxic stress. This observation is consistent with previous reports showing that apoptosis-related pathways contribute to neurotoxicity and that mitochondrial support can improve neuronal cell viability in injury models.^27^^,^^29^

Beyond promoting neuronal survival, astrocytic mitochondrial transfer was associated with a clear improvement in neuronal bioenergetic function. Seahorse extracellular flux analysis showed that exposure to 6-OHDA substantially reduced neuronal oxygen consumption, consistent with impaired mitochondrial respiration. Notably, supplementation with astrocyte-derived mitochondria led to a significant recovery of basal respiration and overall respiratory capacity, even in settings where endogenous astrocytic support was functionally compromised. These observations suggest that the transferred mitochondria are metabolically competent within recipient neurons and contribute meaningfully to the restoration of oxidative metabolism and energy production. Such recovery of respiratory function is particularly relevant in PD, where early bioenergetic deficits are thought to precede synaptic impairment and neuronal loss. In this context, our findings are consistent with the broader importance of mitochondrial dynamics in cellular homeostasis and with evidence that astrocytic mitochondrial regulation can influence neuronal protection in PD-related injury.^30^^,^^31^

One of the most striking outcomes was the impact on neuronal structural plasticity. Sholl analysis revealed that astrocytic mitochondrial infusion increased dendritic branching and mean dendritic intersections in injured neurons (Figures 5A–5C). These morphological improvements suggest that mitochondrial rescue supports not only neuronal survival and bioenergetic recovery, but also structural features required for neuronal connectivity. These findings reinforce earlier reports that astrocytes contribute to synaptogenesis and synaptic stabilization through secreted proteins and other support mechanisms,^3^^,^^8^ and further suggest that mitochondrial delivery may represent an additional mechanism by which astrocytes promote neuronal recovery.

Importantly, these cellular and structural findings were further supported *in vivo* by restoration of dopaminergic integrity in the SNpc. In the 6-OHDA-lesioned rat model, stereotactic delivery of astrocytic mitochondria significantly restored TH immunoreactivity compared with 6-OHDA-lesioned animals, indicating preservation of the dopaminergic neuronal phenotype (Figures 7A and 7B). This *in vivo* TH recovery extends the neuroprotective findings to the 6-OHDA rat model and supports further investigation of astrocytic mitochondrial delivery as a potential therapeutic approach.

We then evaluated whether the observed cellular and molecular changes translated into functional improvement by conducting a series of behavioral tests in 6-OHDA-lesioned rats. As expected, these animals exhibited classic motor deficits, including reduced locomotor activity and impaired rotarod performance. However, animals receiving astrocytic mitochondria showed marked recovery in both motor domains (Figures 8A–8C). Furthermore, significant improvements were observed in non-motor symptoms such as social withdrawal and anxiety-like behavior, as assessed through the social interaction and light/dark box tests (Figures 8D and 8E). These findings are consistent with previous studies showing that mitochondrial transplantation can improve mitochondrial bioenergetics, tissue protection, or functional outcomes across models of ischemic and neurodegenerative injury, although the extent of long-term functional recovery varies between models.^11^^,^^18^^,^^19^^,^^20^^,^^25^

Several studies have previously demonstrated that mitochondrial transplantation or mitochondrial transfer-based interventions confer neuroprotection in experimental models of PD, reinforcing the concept that restoration of mitochondrial function represents a viable therapeutic strategy in neurodegenerative disorders.^23^^,^^24^^,^^25^ While these studies primarily rely on exogenously supplied mitochondria or engineered delivery approaches, the present work extends this concept by providing evidence that astrocytes may serve as an endogenous source of mitochondrial material capable of supporting neuronal recovery under neurotoxic conditions relevant to PD. By integrating mitochondrial depletion, donor mitochondrial perturbation, extracellular mitochondrial detection, add-back rescue, and behavioral outcomes, our study extends the existing mitochondrial transfer paradigm toward a glia-driven reparative mechanism. This astrocyte-centered framework highlights an underappreciated dimension of neuron-glia communication and provides a rationale for further investigation of astrocyte-derived mitochondria in organelle-based therapeutic strategies.

In summary, our findings indicate that astrocyte-derived mitochondrial transfer may contribute to neuronal recovery under neurotoxic conditions. Across *in vitro* and *in vivo* experiments, astrocytic mitochondrial delivery was associated with reduced oxidative stress and apoptosis, improved MMP, respiration and calcium homeostasis, preservation of neuronal structural integrity and dopaminergic markers, and improved motor and non-motor behavioral outcomes. Mitochondrial labeling, extracellular mitochondrial detection, mitochondrial depletion, and add-back experiments provide convergent evidence consistent with astrocyte-to-neuron mitochondrial transfer, although the precise mechanisms of release, uptake, and intracellular integration remain to be established. Together, these findings support the therapeutic potential of astrocyte-derived mitochondrial delivery and warrant further investigation of this approach as a potential cell-free therapeutic strategy for PD.

### Limitations of the study

Although our findings provide evidence for astrocyte-to-neuron mitochondrial transfer and its neuroprotective effects, the mechanisms that control this process require further investigation. In particular, the molecular signals that trigger mitochondrial release from astrocytes and regulate their uptake by injured neurons remain to be identified. The intracellular fate of the transferred mitochondria, including their integration with the neuronal mitochondrial network, functional stability, and long-term persistence, also needs to be examined in greater detail. Future studies should determine whether specific neuronal injury signals guide mitochondrial transfer and identify the pathways involved in maintaining mitochondrial quality after uptake. Further work in chronic PD models will also help establish the optimal dose, timing, and delivery method for astrocyte-derived mitochondria. Addressing these questions will provide a clearer understanding of the transfer process and support the further development of astrocytic mitochondria as a cell-free therapeutic approach.

## Resource availability

### Lead contact

Requests for further information on resources and reagents should be directed to and will be fulfilled by the lead contact, Dr. Shubha Shukla (shubha_shukla@cdri.res.in).

### Materials availability

This study did not generate new unique reagents.

### Data and code availability

All data reported in this paper will be shared by the lead contact upon request. This paper does not report original code. Any additional information required to reanalyze the data reported in this paper is available from the lead contact upon request.

## Acknowledgments

The authors sincerely thank Mr. M. L. Vishwakarma for his assistance with the FACS analysis of the samples during the entire course of the study and Dr. Upendra Kumar Soni for his valuable inputs. Additionally, the authors would like to thank the CSIR-CDRI confocal team for its assistance in confocal microscopy imaging.

This work was supported by funding from CSIR-Phytopharmaceutical Mission Phase-2 (HCP-0038), Phase-3 (MMP075201), and was partially supported by the 10.13039/100031783CSIR-National Laboratories Scheme under the ULIP sub-scheme (MLP002605). Authors express their profound gratitude to Dr. Radha Rangarajan, Director, CSIR-CDRI, for her unwavering support and encouragement. The 10.13039/501100001412Council of Scientific and Industrial Research (CSIR), New Delhi, India, provided research fellowships to J.S. and V.T. Meanwhile, A.S. was supported by a research grant from the 10.13039/501100001501University Grants Commission (UGC), New Delhi, India.

## Author contributions

Conceptualization of the hypothesis, methodology and methods standardization, investigation and execution of most experiments, data analysis, resources (articles), software handling, validation of experiments, and writing – original draft, J.S.; formal analysis, behavioral software setup, and analysis, V.T.; methodology and investigation related to animal behavioral studies and animal handling, A.S.; conceptualization, data verification, manuscript draft editing, reviewing, and commenting, S.S. All authors reviewed and approved the final manuscript.

## Declaration of interests

The authors declare no competing interests.

## Declaration of generative AI and AI-assisted technologies in the manuscript preparation process

During the preparation of this work, the author(s) used Grammarly for grammar and language correction. No generative AI or AI-assisted technologies were used to create figures or to write the manuscript text. After using this tool, the author(s) reviewed and edited the content as needed and take full responsibility for the content of the publication.

## STAR★Methods

### Key resources table

**Table:** 

REAGENT or RESOURCE	SOURCE	IDENTIFIER
**Antibodies**		

Mouse anti-MAP2 antibody, clone AP20	MilliporeSigma	Cat# MAB3418; RRID: AB_94856
Mouse anti-tyrosine hydroxylase (TH) antibody, clone LNC1	MilliporeSigma	Cat# MAB318; RRID: AB_2201528
Rabbit anti-COX4 antibody	Invitrogen	Cat# PA5-29992; RRID: AB_2547466
Goat anti-rabbit IgG (H + L), Alexa Fluor 488	Invitrogen	Cat# A-11008; RRID: AB_143165
Goat anti-mouse IgG (H + L), Alexa Fluor 488	Invitrogen	Cat# A-11001; RRID: AB_2534069
Goat anti-rabbit IgG (H + L), Alexa Fluor 594	Invitrogen	Cat# A-11012; RRID: AB_2534079

**Chemicals, peptides, and recombinant proteins**		

DMEM/F-12	Gibco	Cat# 11320–033
Neurobasal Medium	Gibco	Cat# 21103–049
B-27 Supplement (50X)	Gibco	Cat# 17504–044
GlutaMAX Supplement	Gibco	Cat# 35050061
Penicillin-streptomycin (100X)	Gibco	Cat# 15140–122
Hanks’ Balanced Salt Solution (HBSS)	Gibco	Cat# 14175–095
6-Hydroxydopamine (6-OHDA)	Sigma-Aldrich	Cat# H4381
Mitochondrial division inhibitor 1 (mdivi-1)	Sigma-Aldrich	Cat# M0199
Oligomycin	Sigma-Aldrich	Cat# O4876
FCCP	Sigma-Aldrich	Cat# C2920
Rotenone	Sigma-Aldrich	Cat# R8875
Papain	Sigma-Aldrich	Cat# P4762
Trypsin-EDTA (0.25%), 1X, phenol red	Gibco	Cat# 25200072
Paraformaldehyde	Sigma-Aldrich	Cat# P4707
Poly-L-lysine solution	Sigma-Aldrich	Cat# P6407
Poly-D-lysine hydrobromide	Sigma-Aldrich	Cat# P0899
Fluo-4 AM	Invitrogen	Cat# F14201
MitoSOX Red Mitochondrial Superoxide Indicator	Invitrogen	Cat# M36007
DCFDA (DCFH-DA)	Sigma-Aldrich	Cat# D6883
JC-1	Invitrogen	Cat# T3168
MitoTracker Green FM	Invitrogen	Cat# M7514
MitoTracker Red CMXRos	Invitrogen	Cat# M7512
CellLight Mitochondria-GFP, BacMam 2.0	Invitrogen	Cat# C10600

**Critical commercial assays**		

Dead Cell Apoptosis Kit with Annexin V FITC and propidium iodide (PI)	Invitrogen	Cat# V13242
Pierce BCA Protein Assay Kit	Thermo Scientific	Cat# 23225
Seahorse XF Cell Mito Stress Test Kit	Agilent	Cat# 103334–100
Mammalian Mitochondria Isolation Kit	BioVision	Cat# K288-50

**Experimental models: Organisms/strains**		

Sprague-Dawley rats (neonatal pups and adult males)	CSIR-CDRI animal facility	N/A

**Software and algorithms**		

GraphPad Prism, versions 8.0 and 11.0	GraphPad Software	RRID: SCR_002798; https://www.graphpad.com
ImageJ	National Institutes of Health	RRID: SCR_003070; https://imagej.nih.gov/ij

**Other**		

Transwell-Clear inserts, PET membrane, 12 mm diameter, 3.0 μm pore size	Corning	Cat# 3462
FACSCalibur flow cytometer	BD Biosciences	FACSCalibur
FACSAria cell sorter	BD Biosciences	FACSAria

### Experimental model and study participant details

#### Animals

Adult male Sprague-Dawley (SD) rats weighing 180–200 g (7–8 weeks old) were obtained from the National Laboratory Animal Center (NLAC) at the CSIR-Central Drug Research Institute (CDRI), Lucknow, India. Animals were housed under controlled environmental conditions with a 12-h light/dark cycle (lights on from 8:00 a.m. to 8:00 p.m.), a temperature of 24 ± 1 °C, and *ad libitum* access to standard food and water. All rats were acclimatized to the housing environment for 5 days prior to the start of the experiments.

Following acclimatization, animals were randomly assigned to the experimental treatment groups. Treatment preparation and administration were performed by an investigator who was not involved in subsequent outcome assessment. Behavioral outcome assessments were performed by investigators blinded to treatment-group allocation.

Primary cortical neurons and astrocytes were cultured from neonatal SD rat pups (postnatal day 0–1), weighing 5–20 g. Pups were allowed to feed once from the dam prior to separation for culture preparation. The sex of the neonatal pups was not determined before culture preparation. Because the *in vivo* experiments used only adult male rats and the sex of the neonatal pups used for primary cultures was not determined, sex-related differences were not evaluated in this study. Detailed culture procedures are provided under Method details.

#### Ethical approval

All animal procedures were conducted in strict accordance with the guidelines of the Committee for the Purpose of Control and Supervision of Experiments on Animals (CPCSEA), Government of India, and were approved by the Institutional Animal Ethics Committee (IAEC) of CSIR-CDRI under approval number IAEC/2019/55/REN. Experimental protocols adhered to the ethical standards outlined by the Indian National Science Academy (INSA) for the care and use of laboratory animals, in line with internationally accepted norms.

### Method details

#### *In vitro* studies

##### Transwell co-culture assay

To test our hypothesis, we established a transwell co-culture system in which primary neurons were seeded in the lower chamber and astrocytes in the upper insert. Transwell inserts with a pore size of 3.0 μm (Cat. No. 3462; 12 mm diameter, Corning, USA) were used to permit the exchange of organelles or extracellular particles between the two cell types.

##### Primary neuron culture

Primary cortical neurons were isolated and cultured from 0–1-day-old Sprague-Dawley (SD) rat pups following previously established protocols.^32^ Cerebral cortices were gently dissociated in 1× Hanks’ Balanced Salt Solution (HBSS; Cat. No. 14175-095; Gibco) and enzymatically digested with 0.1% papain at 37 °C for 20 min. After enzymatic treatment, the tissue was subjected to 10–15 gentle triturations to obtain a single-cell suspension. The dissociated neurons were then resuspended in neurobasal medium (NBM) supplemented with 2% B27 (Invitrogen, USA) and 0.5 mM GlutaMAX (Gibco, USA) and plated at a density of 0.5 × 10^6^ cells/well in 12-well plates pre-coated with 0.1 mg/mL poly-L-lysine. Cells were incubated at 37 °C in a humidified atmosphere containing 5% CO_2_ and 95% air. Neuronal cultures were maintained by replacing 50% of the culture medium with fresh, pre-warmed NBM every third day until the neurons reached maturation.

#### Primary astrocyte culture

Primary astrocyte cultures were prepared from the cerebral cortices of neonatal rat pups following previously established protocols, with minor adaptations, as described by Hayakawa et al. (Nature, 2016).^15^ Prior to dissection, 30 mL of astrocyte culture medium [DMEM/F-12 supplemented with 10% heat-inactivated fetal bovine serum (FBS) and 1% penicillin/streptomycin] was pre-warmed to 37 °C. T-75 culture flasks were coated with 10 mL of 50 μg/mL poly-D-lysine (PDL) prepared in DEPC-treated water for 2 h.

All dissections were performed under sterile conditions. Hanks’ Balanced Salt Solution (HBSS) was used throughout the procedure, with pH maintained between 7.2 and 7.4. Neonatal pups were euthanized by decapitation, after which cerebral cortices were carefully dissected, minced, and transferred into HBSS buffer.

Following removal of HBSS, tissue was incubated with 0.25% Trypsin-EDTA (Cat. No. 25200072; Gibco) and enzymatically digested for 30 min at 37 °C in a water bath. The digested tissue was pelleted by centrifugation at 300 × g for 5 min, and the supernatant was discarded. The cell pellet was resuspended in 10 mL of astrocyte plating medium and gently triturated 10–15 times using a 10 mL pipette to obtain a single-cell suspension. The suspension was then passed through a 70 μm cell strainer to remove undissociated tissue fragments. After aspirating the PDL solution and washing the coated flasks with HBSS, cells were plated at a density of approximately 6 × 10^5^ cells/cm^2^ into the prepared T-75 flasks.

Culture medium was replaced every 48 h. Upon reaching confluency, typically between days 7–8 *in vitro*, flasks were placed on an orbital shaker at 180 rpm for 30 min to remove loosely attached microglia. The supernatant was discarded and replaced with fresh medium (20 mL). To further eliminate oligodendrocyte precursor cells (OPCs), flasks were subjected to an additional shaking step at 240 rpm for 6 h. The resulting purified astrocyte monolayer was washed twice with PBS and maintained in fresh culture medium at 37 °C in a humidified CO_2_ incubator until further use.

#### Preparation of astrocyte-conditioned medium (ACM) and mitochondrial-depleted astrocyte-conditioned medium (md-ACM)

*Astrocyte-conditioned medium (ACM)* was prepared using a protocol adapted from Hayakawa et al. (Nature, 2016),^15^ with minor modifications optimized for our experimental system. Primary astrocytes were grown to approximately 70–80% confluency and gently washed twice with phosphate-buffered saline (PBS) to remove residual serum components. Cells were then incubated in serum-free Neurobasal medium for 24 h at 37 °C under standard culture conditions. Following incubation, the conditioned medium was collected and processed sequentially to remove cellular contaminants. First, the medium was centrifuged at 300 × g for 5 min at 4 °C to eliminate detached cells. The supernatant was then transferred to a fresh tube and centrifuged at 2,000 × g for 10 min at 4 °C to further clear cellular debris and large fragments.

To generate *mitochondrial-depleted astrocyte-conditioned medium* (md-ACM), the clarified ACM was subjected to high-speed centrifugation at 10,000 × g for 20 min at 4 °C, a step designed to pellet mitochondria-sized extracellular particles. The resulting supernatant was subsequently passed through a 0.2 μm pore-size syringe filter (Millipore) to remove remaining large extracellular structures. This centrifugation and filtration-based approach has been previously demonstrated to effectively deplete functional extracellular mitochondria from astrocyte-conditioned media. Because this centrifugation and filtration-based procedure is not selective for mitochondria, depletion of other particulate or large extracellular components cannot be excluded.

Freshly prepared ACM or md-ACM was used immediately for neuronal treatments in all experiments to avoid potential loss of labile extracellular components.

#### Mdivi-1 pretreatment of donor astrocytes

To pharmacologically perturb donor mitochondrial function prior to isolation, primary astrocytes were treated with mdivi-1 (50 μM) for 12 h under standard culture conditions. Following pretreatment, cells were washed with PBS and used for the indicated experiments. mdivi-1 was used as a pharmacological tool to perturb mitochondrial function; however, its effects are not necessarily restricted to mitochondrial fission, and therefore it was not used to infer a fission-specific mechanism.

#### Flow-cytometric measurement of intracellular ROS

ROS generation was measured using the DCFH-DA dye (Cat. No. D6883; Sigma-Aldrich, St. Louis, MO, USA) as described before.^33^ The cell-permeable probe DCFH-DA is a non-fluorescent compound that readily diffuses into cells, where intracellular esterases hydrolyze it to yield DCFH, a non-fluorescent and membrane-impermeant intermediate. Once trapped within the cytoplasm, DCFH remains relatively stable for several hours. In the presence of reactive oxygen species (ROS), DCFH undergoes oxidation to form 2′,7′-dichlorofluorescein (DCF), a highly fluorescent molecule. Upon excitation at 488 nm, DCF emits green fluorescence, the intensity of which is directly proportional to intracellular ROS levels. Thus, changes in DCF fluorescence serve as an indicator of oxidative stress within cells. In this study, neuronal cultures were incubated with 10 μM DCFH-DA for 30 min at 37 °C under dark conditions. After incubation, cells were washed and resuspended in phosphate-buffered saline (PBS), followed by immediate analysis using flow cytometry (FACSCalibur, Becton Dickinson, San Jose, CA, USA). For all flow cytometry experiments, at least 10,000 events were acquired per sample, and population frequencies were expressed as percentages of gated cells.

#### Flow-cytometric measurement of mitochondrial ROS

Neurons from each experimental group were incubated with MitoSOX Red (Invitrogen; Cat. No. M36007) at a final concentration of 500 nM for 30 min at 37 °C, protected from light. Cells were washed three times with warm buffer and analyzed by flow cytometry using a BD FACSCalibur.

#### Measurement of mitochondrial membrane potential

The mitochondrial membrane potential in primary neurons was detected using JC-1 dye (Cat. No. T3168; Invitrogen, USA) in order to measure the health of mitochondria. JC-1 is a cationic dye widely used to assess mitochondrial membrane potential (MMP),^34^ as its fluorescence properties shift in response to changes in polarization. In depolarized or low potential mitochondria, JC-1 remains in its monomeric form, emitting green fluorescence (excitation/emission: 514/529 nm). However, under high membrane potential conditions, JC-1 aggregates into J-aggregates, leading to a fluorescence shift toward red (excitation/emission: 585/590 nm). This transition reflects increased mitochondrial polarization and is characterized by a red to green fluorescence ratio that serves as a reliable indicator of mitochondrial health. Thus, a shift from green to orange-red fluorescence corresponds to more polarized, functionally active mitochondria. For the experiments, neurons were incubated with JC-1 at 1 μg/mL for 30 min at 37 °C, and green and red fluorescence were measured using a BD FACSCalibur flow cytometer.

#### Measurement of early apoptosis

Annexin V conjugated to fluorescent probes is widely utilized for the identification of apoptotic cells due to its strong affinity for phosphatidylserine (PS), an anionic phospholipid. Annexin V is a 35–36 kDa human calcium-dependent phospholipid-binding protein that specifically recognizes PS in the presence of Ca^2+^ ions. In healthy cells, PS is predominantly confined to the inner leaflet of the plasma membrane. However, during early apoptosis, structural changes in the membrane result in externalization of PS to the outer leaflet, where it becomes accessible to Annexin V binding. To assess apoptosis, we employed the Dead Cell Apoptosis Kit with Annexin V FITC and propidium iodide (PI) (Cat. No. V13242; Invitrogen, USA). The experiment was conducted according to the manufacturer’s instructions provided by Invitrogen (Molecular Probes, USA). Semi-confluent (60% confluency) cells were seeded in 6-well plates and treated with 6-OHDA for 24 h. Trypsinized cells were spun at 900 rpm for 5 min to extract the pellets. Annexin V and PI were added when the cells were resuspended in 1× Annexin binding buffer. The BD FACSCalibur apparatus (BD Biosciences, USA) was utilized to collect and analyze apoptotic events.

#### Mitochondrial stress assessment

The oxygen consumption rate (OCR),^35^ an indicator of mitochondrial respiration, was measured using an Agilent Seahorse XFp Analyzer. XFp cell culture miniplates were seeded with 20,000 cells per well a day prior to the experiment. Before seeding, neurons were dissociated using 0.25% Trypsin-EDTA. Each treatment was tested in three replicate miniplate wells (technical triplicates). To the XF Assay Media, 5.5 mM glucose, 1 mM sodium pyruvate, and 2 mM L-glutamine were added. The following inhibitor final doses were used for the mitochondrial stress test: 2.0 μM oligomycin, 1.5 μM FCCP, and 2.0 μM rotenone. Following the addition of each inhibitor to the media, three measurements were taken in sequence. OCR traces and mitochondrial stress test parameters were generated using the Agilent Seahorse analysis software according to the manufacturer’s instructions (Seahorse XF Cell Mito Stress Test Kit; Agilent, Cat. No. 103334-100). For the assay, neurons were cultured and analyzed directly in XFp cell-culture miniplates; therefore, the transwell co-culture system was not used. Experimental conditions included untreated neurons, 6-OHDA treated neurons, 6-OHDA treated neurons exposed to md-ACM, 6-OHDA treated neurons directly treated with mdivi-1, and the corresponding mdivi-1 or md-ACM-treated groups supplemented with isolated astrocytic mitochondria.

#### Measurement of intracellular Ca^2+^ levels

The manufacturer’s methodology was used to determine the intracellular Ca^2+^ level.^34^ In brief, cells at a density of 5x10^4^ cells per well were first treated for 30 min at 37 °C in the dark with 5 μM Fluo-4 AM (Cat. No. F14201, Invitrogen USA). Following treatment, cells were washed with PBS to eliminate extracellular Fluo-4 AM and resuspended in PBS. The fluorescence signal was detected using a laser system (FACSCalibur; BD Biosciences, USA). An argon laser was used to activate Fluo-4 at 494 nm, and the emission was observed at 506 nm. The fluorescence intensity for each sample was calculated using CellQuest software (BD Biosciences, USA).

#### Mitochondrial mass analysis

MitoTracker dye (MitoTracker Green FM, Cat. No. M7514, Invitrogen USA), which passively diffuses along the plasma membrane and aggregates in active mitochondria, was applied to neurons and astrocytes to verify mitochondrial number or quantification. Because these cells were co-cultured and grown in culture plates, three replicates were used for each condition. When the cells had reached the necessary confluency, the medium was removed from the plate, and a preheated (37°C) staining solution containing the 50–100 nM MitoTracker Green FM was added. After 15 to 20 min of incubation, cells were trypsinized with 0.25% trypsin from Invitrogen USA and centrifuged at 900 rpm for 5 min. Phosphate buffer saline (PBS) was used to wash the cells twice, and they were then placed on ice before being subjected to flow cytometry analysis (FACSCalibur, Becton Dickinson, San Jose, CA).

#### Mitochondrial labeling using MitoTracker red CMXRos

The MitoTracker Red CMXRos probe is employed in living cells to label mitochondria, and its accumulation is influenced by membrane potential (functionally activated mitochondria). It also retains well after aldehyde fixation. Neurons/astrocytes were incubated with MitoTracker Red CMXRos (Cat. No. M7512, Invitrogen USA) at a concentration of 50 nM before being co-cultured and grown in a 12-well plate (culture treatment) with the appropriate culture medium in three replicates for each condition. The cells were then treated for 24 h with MitoTracker Red CMXRos before being rinsed with PBS and fresh medium was added for live cell imaging using a DMI6000 Leica inverted fluorescent microscope combined with a digital CCD camera with a 10x or 20x magnification (Leica, Wetzlar, Germany). Flow cytometry (FACSAria, Becton Dickinson, San Jose, CA) was used to perform several experiments, including cell sorting and mean fluorescence intensity (MFI) of a tracking dye.

#### Mitochondrial labeling using mito-GFP

BacMam 2.0, CellLight Mitochondria-GFP, simplifies the process of labeling mitochondria with green fluorescent protein (GFP) in living cells. This commercially available construct expresses GFP coupled to the E1α pyruvate dehydrogenase leader sequence and is transfected into cells using BacMam 2.0 technology, allowing for the monitoring of mitochondria-GFP mobility in live cells independent of mitochondrial membrane potential (MMP). CellLight Mitochondria-GFP, BacMam 2.0 (Cat. No. C10600, Invitrogen USA) was treated with astrocytes. These cells were grown in a 12-well plate with the suitable culture medium in three replicates for each condition. After 24 h of treatment with the Mitochondria-GFP construct (10 μL per 10,000 cells), the cells were washed with PBS and fresh medium was added for live cell microscopy and FACS. Cell sorting was carried out utilizing flow cytometry using this construct (FACSAria, Becton Dickinson, San Jose, CA) and live microscopic studies using a DMI6000 Leica inverted fluorescent microscope combined with a digital CCD camera with a 10x or 20x magnification (Leica, Wetzlar, Germany).

#### Isolation of mitochondria from primary astrocytes

Mitochondria are the powerhouse of the cell since they provide the majority of the energy in the form of adenosine triphosphate (ATP). Isolated mitochondria are the most commonly studied sample for mitochondrial respiration, respiratory complex assembly, apoptosis, mtDNA and mtRNA, and mitochondrial protein profiling. For tissue and cultured cells, we used the Mammalian Mitochondria Isolation Kit (Cat. No. K288-50; BioVision, USA). After collecting a pellet of about 2 x10^7^ cells, 100 μL of mitochondria isolation buffer was added before vortexing for 5 s and incubating on ice for 2 min 100 μL of Reagent A was added and incubated for 5 min on ice before centrifuging for 10 min at 600 x g at 4 °C. Supernatant was collected in another tube and centrifuged at 7,000 x g for 10 min at 4°C. The pellet was rinsed in mitochondria isolation buffer after the supernatant was removed. The mitochondrial pellet was resuspended in mitochondrial storage buffer.

#### Immunocytochemistry

Immunocytochemistry (ICC) is a semi-quantitative method used to evaluate the distribution, morphology, and subcellular localization of specific antigens within cultured cells.^34^ For ICC analysis, cells cultured on coverslips were fixed with ice-cold 4% paraformaldehyde in phosphate-buffered saline (PBS) for 10 min to preserve cellular architecture. Following fixation, non-specific binding was blocked by incubating the cells for 45 min at room temperature in a blocking solution containing 1% bovine serum albumin (BSA) and 0.3% Triton X-100 in PBS. Primary antibodies were diluted in the same blocking buffer and applied to the cells overnight at 4 °C. The following day, cells were washed with PBS and incubated with fluorophore-conjugated secondary antibodies (Invitrogen, USA) for 2 h at room temperature. After final washes, coverslips were prepared for imaging. Subsequently, coverslips were mounted using ProLong Gold Antifade Mountant (Invitrogen, USA) and examined under an inverted fluorescence microscope (Leica DMI 6000). Details of the primary antibodies used for ICC analysis are provided in the key resources table.

#### Sholl analysis

Sholl analysis was performed to assess dendritic complexity and arborization, following the methodology described by Ferreira et al.^36^ The analysis was conducted using the Sholl Analysis plugin integrated into the ImageJ software (NIH). Maximum intensity projection images of individual neurons were used for semi-automated tracing. Concentric circles centered on the soma were generated at fixed radial intervals, and the number of dendritic intersections with each circle was quantified. This allowed for a systematic evaluation of dendritic branching and growth radiating from the neuronal soma.

#### Animal studies

##### 6-OHDA lesion surgery

The 6-hydroxydopamine (6-OHDA) lesion model was established following a previously described protocol.^37^ Briefly, adult Sprague-Dawley rats were anesthetized using isoflurane and securely positioned in a Stoelting stereotaxic apparatus (Wood Dale, IL, USA). A 30-gauge Hamilton syringe (Hamilton Corporation, Switzerland) was used to unilaterally inject 16 μg of 6-OHDA, dissolved in normal saline, into the right medial forebrain bundle. The stereotaxic coordinates relative to bregma were: anterior to posterior (AP) = −4.4 mm, medial to lateral (ML) = +1.2 mm, and dorsal to ventral (DV) = −7.8 mm. The injection was performed at a flow rate of 0.5 μL/min. To minimize backflow along the needle track, the syringe was left in place for an additional 5 min after injection before being slowly withdrawn.

#### Stereotactic delivery of mitochondria

Following previously reported methods with minor modifications,^11^^,^^20^ rats were anesthetized using isoflurane, and their scalps were shaved prior to placement in a stereotaxic frame (Stoelting, Wood Dale, IL, USA). Under aseptic conditions, a midline incision was made using fine surgical scissors, and the scalp was retracted to expose anatomical landmarks including the bregma and lambda. A small burr hole was drilled at the predetermined stereotaxic coordinates over the substantia nigra pars compacta (SNpc) region. A 10 μL Hamilton syringe containing the mitochondrial suspension was carefully positioned at the target site.

A total of 5 μL of mitochondrial suspension, containing approximately 1 × 10^5^ mitochondria, was slowly injected into the SNpc at the following stereotaxic coordinates: AP −5.2 mm from bregma, ML +2.0 mm from the midline, and DV −7.6 mm from the dura. This stereotaxic infusion was performed on three separate occasions on days 0, 7, and 14 to ensure sustained mitochondrial delivery. Following the injection, the needle was withdrawn slowly, the scalp incision was sutured, and animals were administered analgesics. Rats were then placed in a pre-warmed recovery chamber and monitored until full recovery.

#### Immunohistochemistry (IHC)

To examine dopaminergic neuronal integrity in the substantia nigra pars compacta (SNpc), immunofluorescence staining was performed on coronal brain sections. Rats were deeply anesthetized with sodium pentobarbital and transcardially perfused with saline followed by 4% paraformaldehyde in phosphate buffer. Brains were removed, post-fixed overnight, cryoprotected through graded sucrose solutions, and sectioned coronally at 35 μm. Free-floating sections spanning the SNpc were washed three times in Tris-buffered saline (TBS), followed by blocking and permeabilization. Sections were incubated overnight at 4 °C with mouse anti-tyrosine hydroxylase antibody (Cat. No. MAB318; MilliporeSigma; 1:1000). After thorough washing in TBS, sections were incubated with fluorescence-conjugated secondary antibodies (1:400, Invitrogen). Following additional washes, nuclei were counterstained with DAPI. Images were acquired using a Leica DMI6000 fluorescence microscope under identical acquisition settings, and TH immunoreactivity within the SNpc was quantified using ImageJ software.^38^

#### Open-field test

Locomotor activity was assessed using the OptoVarimax open-field monitoring system (OptoM-3: Columbus Instruments, USA). Each rat was individually placed in a transparent locomotor chamber measuring 42.5 cm × 42.5 cm to evaluate spontaneous activity. The system recorded both horizontal ambulation and vertical rearing behaviors through infrared beam breaks, providing quantitative measurements of exploratory and motor activity in an open-field environment. Prior to testing, rats were habituated in the open-field arena for 10 min to minimize novelty-induced stress. Locomotor activity was then recorded for a 30-min period. Horizontal movement was quantified as total distance traveled (XY ambulatory), while vertical exploratory behavior was assessed by measuring rearing events (Z-counts). To eliminate potential olfactory cues and prevent behavioral interference between trials, the arena was thoroughly cleaned with 10% ethanol between each animal.^38^

#### Rotarod test

Neuromuscular coordination and motor performance were assessed using a rotarod apparatus (Rotamax-5; Columbus Instruments, USA) with an accelerating speed ranging from 5 to 20 rpm over a 5-min testing period. Prior to 6-OHDA lesioning, all animals underwent a three-day training phase to ensure baseline competency on the rotating rod, with a target performance of 300 s. The final motor coordination test was conducted on day 28 post-surgery using the same acceleration protocol. The latency to fall, defined as the time (in seconds) the animal remained on the rod before falling, was recorded as the primary outcome measure.^38^

#### Light/dark test

The light/dark (LD) test was used to assess anxiety-like behavior in rodents and is based on the conflict between the tendency to explore a novel environment and the aversion to brightly illuminated areas.^38^ Crawley and colleagues described this paradigm as a variant of Montgomery’s model.^39^ The apparatus consisted of a polypropylene chamber (44 × 21 × 21 cm), with one-third of the chamber designated as the dark compartment and the remaining area as the light compartment. Individual SD rats were placed in the apparatus and allowed to explore freely for 10 min. Each animal was tested once. The time spent in the light and dark compartments and the number of transitions between the compartments were recorded. Total locomotor activity across both compartments was also measured.

#### Social interaction test

Social behavior was assessed using a three-chambered apparatus, as described by Crawley (2007),^40^ designed to evaluate sociability in rodents. The apparatus consisted of a transparent plexiglass rectangular box (112 cm L × 56 cm W × 40 cm H) without a top. The central compartment (32 cm L × 56 cm W × 40 cm H) was flanked by two identical side chambers (40 cm L each), and all compartments were interconnected by small retractable doorways (11 cm × 10 cm) to allow free movement.

Each side chamber contained a stainless-steel cylindrical wire cage (8 cm diameter × 12 cm height; 1.5 cm bar spacing). During the habituation phase, the experimental rat was placed in the central compartment and allowed to freely explore all three chambers for 10 min. After acclimatization, a novel male Sprague-Dawley rat of the same age was placed under the wire cup in one of the side chambers (designated the “social chamber”), while the opposite chamber contained an empty, identical wire cup (designated the “non-social chamber”).

The test rat was then allowed to explore for an additional 10 min during the sociability phase. Behavior was recorded using a video camera, and the total time spent in active social interactions defined as sniffing, following, or grooming within 2 cm of the wire cage containing the conspecific (unfamiliar) rat was manually scored by two observers blinded to the experimental groups. To prevent olfactory cues from influencing behavior, the apparatus was thoroughly cleaned with 10% ethanol between trials.

#### Amphetamine-induced rotational behavior

To assess nigrostriatal dopaminergic dysfunction, amphetamine-induced rotational behavior was evaluated on day 28 following 6-OHDA lesioning.^38^ Rats received an intraperitoneal injection of D-amphetamine sulfate (5 mg/kg, i.p.), and after a 30-min acclimation period, rotational activity was recorded for 30 min. Ipsilateral and contralateral rotations were counted, and net ipsilateral rotation was calculated as the difference between the number of turns toward the lesioned side and those toward the non-lesioned side over the observation period (Net ipsilateral rotation = ipsilateral turns − contralateral turns in 30 min).

### Quantification and statistical analysis

Data were analyzed using GraphPad Prism software (versions 8.0 and 11.0; GraphPad Software, San Diego, CA, USA). Comparisons between experimental groups were performed using unpaired t-tests, one-way or two-way analysis of variance (ANOVA), as appropriate, followed by Bonferroni post hoc tests to determine significance between multiple groups. All graphical representations were generated using the same software. Statistical significance was defined as a *p*-value less than 0.05. For *in vitro* experiments, *N* = 3 represents three independent biological experiments performed separately under the indicated experimental conditions, unless otherwise stated. Technical replicates, where applicable, were averaged within each independent experiment and were not treated as independent biological replicates. Unless otherwise stated, quantitative data are presented as mean ± SEM, where the mean represents the measure of center and SEM represents the measure of precision. For *in vivo* experiments, *n* = 8 represents eight individual adult male rats per experimental group. The statistical test, exact sample size, and other experiment-specific statistical details are provided in the corresponding figure legends and figures. Statistical significance is indicated as ∗*p* < 0.05, ∗∗*p* < 0.01, ∗∗∗*p* < 0.001, and ∗∗∗∗*p* < 0.0001; ns, not significant.

### Additional resources

This study did not generate additional resources.
