# Supplementary material for: Astrocytic mitochondria restore neuronal bioenergetics and function in Parkinson disease models
Source: iScience. 2026 Nov 6;29(11):118138. doi: 10.1016/j.isci.2026.118138 (PMC13760966; doi:10.1016/j.isci.2026.118138)
Supplement: Supplementary file 1 [file mmc1.pdf]

iScience, Volume *29*

Supplemental information

Astrocytic mitochondria restore neuronal bioenergetics and function in Parkinson disease models

Jitendra Singh, Virendra Tiwari, Alka Sharma, and Shubha Shukla

Supplemental figures

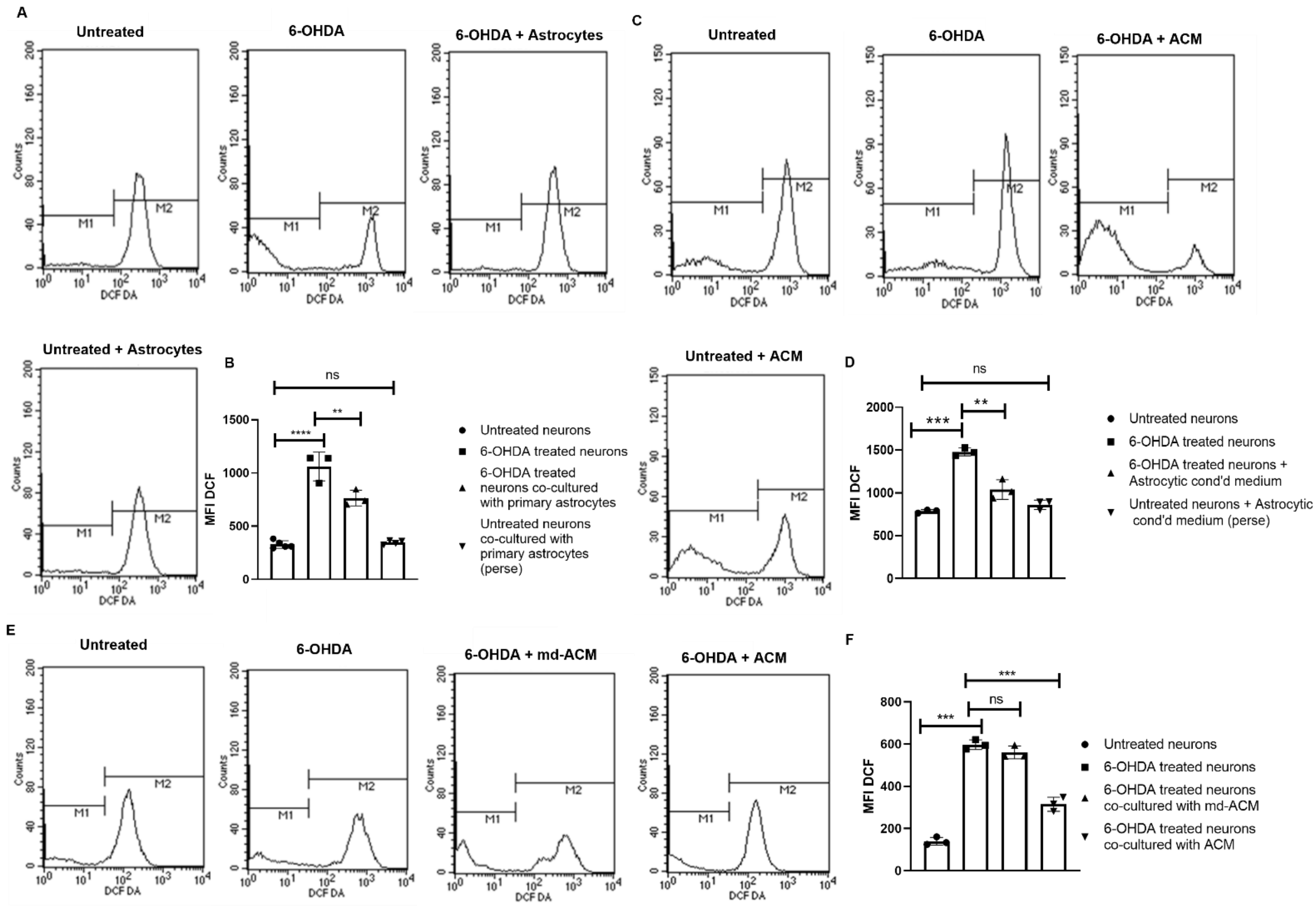

Figure S1. Astrocytic mitochondria reduce ROS levels in 6-OHDA treated neurons, related to Figure 1. FACS analysis showing intracellular ROS generation measured by DCF-DA mean fluorescence intensity (MFI-DCF). Representative FACS histograms show the following experimental conditions: (A) untreated neurons, 6-OHDA treated neurons, 6-OHDA treated neurons co-cultured with astrocytes, and untreated neurons co-cultured with astrocytes; (C) untreated neurons, 6-OHDA treated neurons, 6-OHDA treated neurons cultured in astrocyte-conditioned medium (ACM), and untreated neurons cultured in ACM; and (E) untreated neurons, 6-OHDA treated neurons, 6-OHDA treated neurons cultured with mitochondrial-depleted astrocyte-conditioned medium (md-ACM), and 6-OHDA treated neurons cultured with ACM. Bar graphs (B, D, and F) show corresponding quantification of ROS levels for panels A, C, and E, respectively. Data are presented as mean ± SEM from three independent experiments (N = 3). Statistical analysis was performed using one-way ANOVA followed by Bonferroni post hoc test. Statistical significance is indicated as *P < 0.05, **P < 0.01, ***P < 0.001, and ****P < 0.0001; ns, not significant.

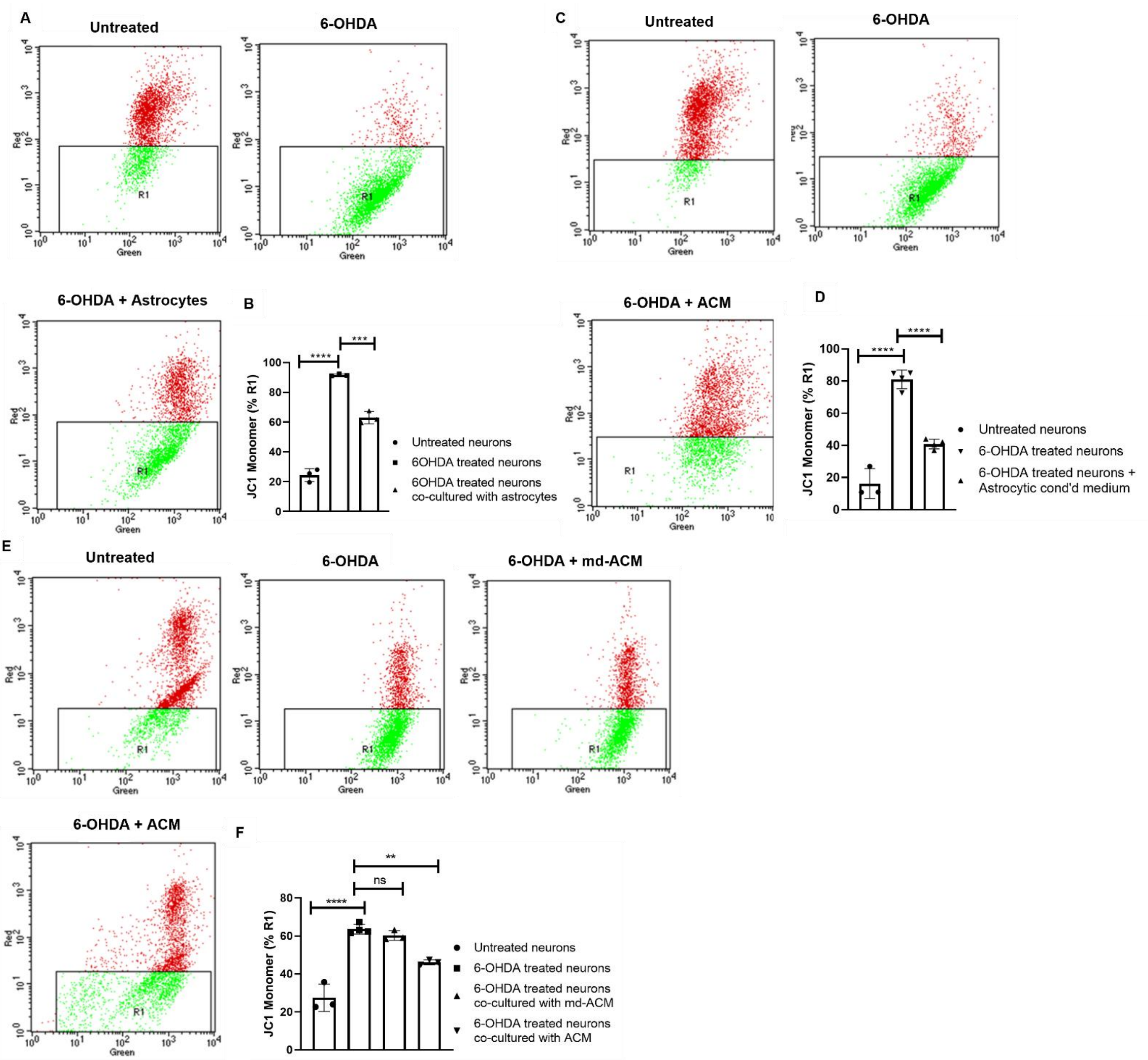

Figure S2. Astrocyte co-culture and conditioned medium improve mitochondrial membrane potential in 6-OHDA treated neurons, related to Figure 1. Flow cytometric analysis showing mitochondrial membrane potential using JC-1 dye. The gated green population represents JC-1 monomers associated with depolarized mitochondria, whereas the red population represents polarized mitochondria. Representative FACS dot plots show the following experimental conditions: (A) untreated neurons, 6-OHDA treated neurons, and 6-OHDA treated neurons co-cultured with astrocytes; (C) untreated neurons, 6-OHDA treated neurons, and 6-OHDA treated neurons cultured in astrocyte-conditioned medium (ACM); and (E) untreated neurons, 6-OHDA treated neurons, 6-OHDA treated neurons cultured with mitochondrial-depleted astrocyte-conditioned medium (md-ACM), and 6-OHDA treated neurons cultured with ACM. Bar graphs (B, D, and F) show the corresponding percentages of JC-1 monomer-positive cells for panels A, C, and E, respectively. Data are presented as mean ± SEM from three independent experiments (N = 3). Statistical analysis was performed using one-way ANOVA followed by Bonferroni post hoc test. Statistical significance is indicated as *P < 0.05, **P < 0.01, ***P < 0.001, and ****P < 0.0001; ns, not significant.

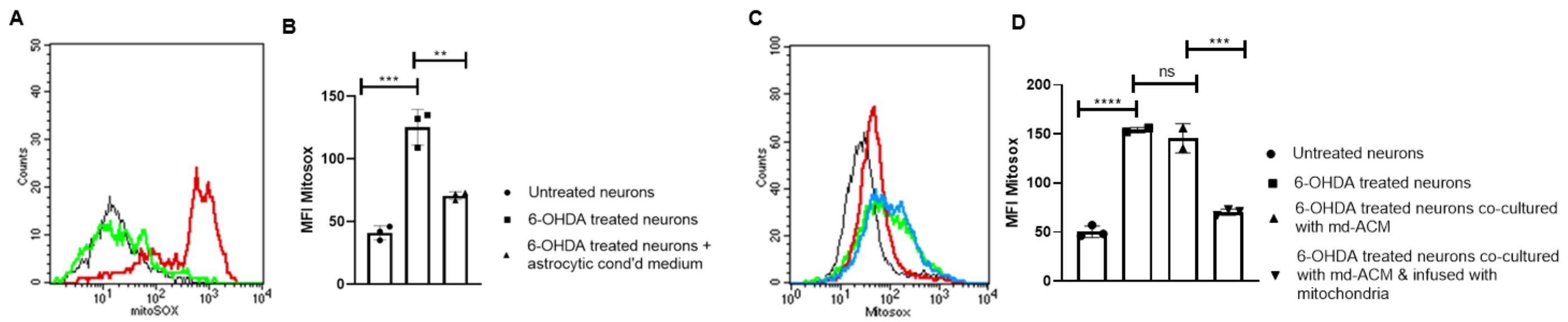

Figure S3. Astrocyte-conditioned medium and mitochondrial add-back reduce mitochondrial ROS in 6-OHDA treated neurons, related to Figure 2. Flow cytometric analysis of MitoSOX fluorescence was used to measure mitochondrial superoxide levels. (A) Representative fluorescence histograms: black line depicts untreated neurons, red line depicts non-co-cultured neurons treated with 6-OHDA. Green line depicts 6-OHDA treated neurons cultured with astrocyte-conditioned medium (ACM). (B) Quantification of MitoSOX mean fluorescence intensity (MFI) for the experimental conditions shown in panel A. (C) Representative fluorescence histograms: black line depicts untreated neurons, blue line depicts neurons treated with 6-OHDA. Green line depicts 6-OHDA treated neurons co-cultured with mitochondrial-depleted astrocyte-conditioned medium (md-ACM). Red line depicts 6-OHDA treated neurons co-cultured with md-ACM and infused with astrocytic mitochondria. (D) Quantification of MitoSOX MFI for the experimental conditions shown in panel C. Quantitative data in panels B and D are presented as mean ± SEM from three independent experiments (N = 3). Statistical analysis was performed using one-way ANOVA followed by Bonferroni post hoc test. Statistical significance is indicated as *P < 0.05, **P < 0.01, ***P < 0.001, and ****P < 0.0001; ns, not significant.

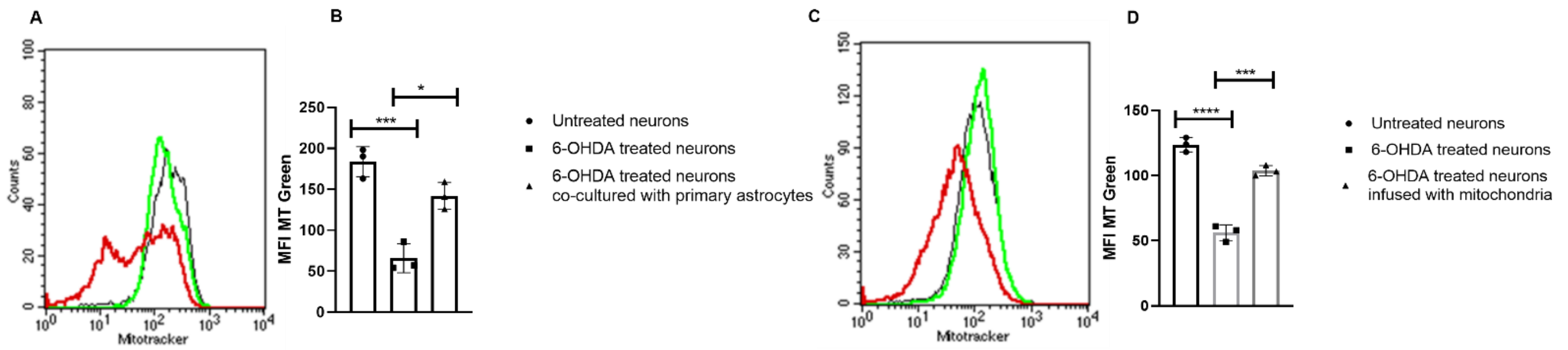

Figure S4. Astrocyte co-culture and mitochondrial delivery restore mitochondrial mass in 6-OHDA treated neurons, related to Figure 3. Flow cytometric analysis showing mitochondrial mass measured as mean fluorescence intensity (MFI) using MitoTracker Green FM. (A) Representative fluorescence histograms: black line depicts untreated neurons, red line depicts non-co-cultured neurons treated with 6-OHDA and green line depicts 6-OHDA treated neurons co-cultured with astrocytes. Bar graph (B) shows quantification of MitoTracker Green FM MFI for the conditions shown in panel A. (C) Representative fluorescence histograms: black line depicts untreated neurons, red line depicts non-co-cultured neurons treated with 6-OHDA and green line depicts 6-OHDA treated neurons infused with mitochondria. Bar graph (D) shows quantification of MitoTracker Green FM MFI for the conditions shown in panel C. Data are presented as mean ± SEM from three independent experiments (N = 3). Statistical analysis was performed using one-way ANOVA followed by Bonferroni post hoc test. Statistical significance is indicated as *P < 0.05, **P < 0.01, ***P < 0.001, and ****P < 0.0001; ns, not significant.

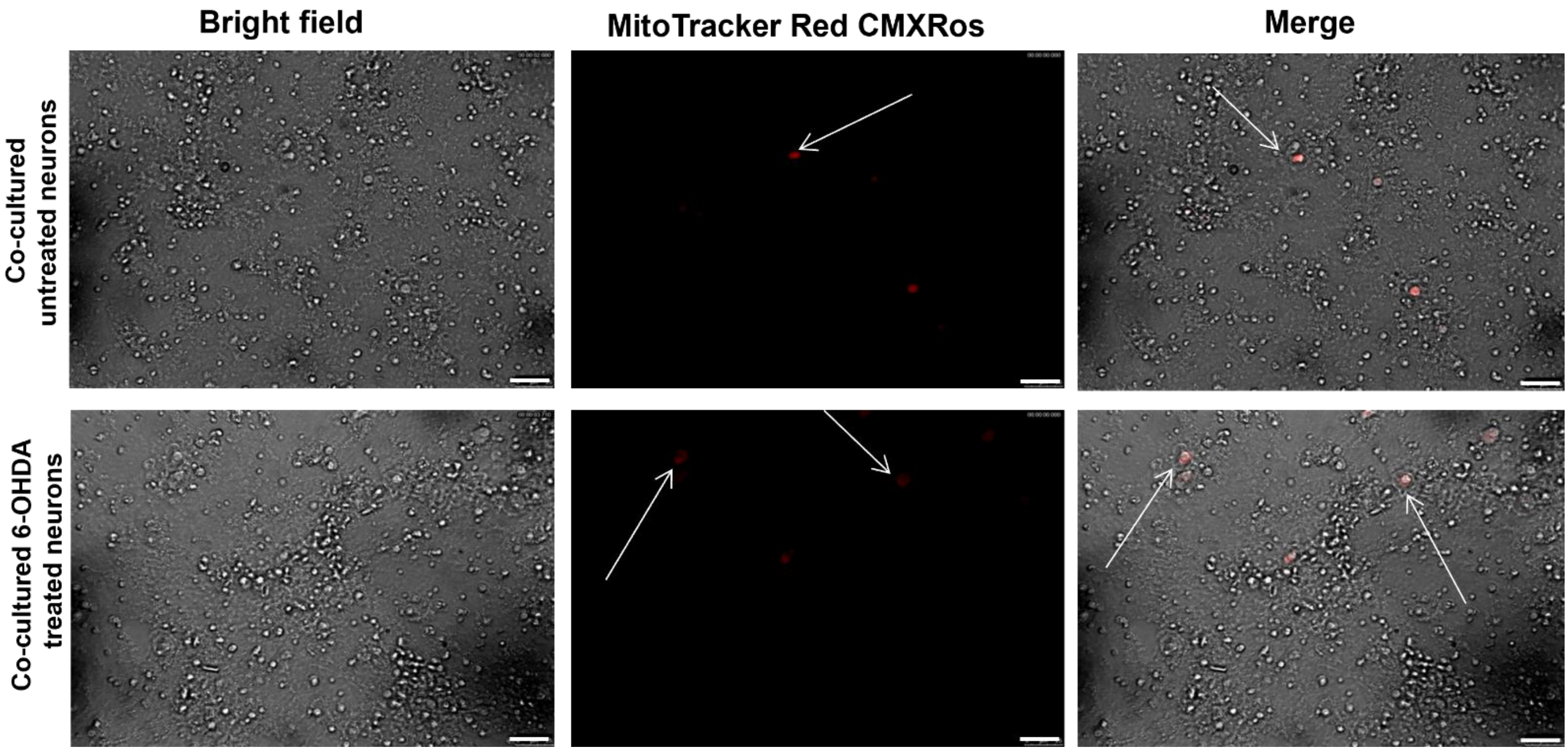

Figure S5. Detection of astrocyte-derived mitochondrial signals in neuronal co-cultures, related to Figure 3. Representative live-cell bright-field and fluorescence images showing astrocyte-derived mitochondria labeled with MitoTracker Red CMXRos in co-cultured untreated neurons (upper row) and co-cultured 6-OHDA treated neurons (lower row). Red fluorescent puncta indicate astrocyte-derived mitochondrial signals associated with neurons, and arrows mark representative mitochondria-positive regions. Images were captured using a Leica DMI6000 microscope. Images are representative of three independent experiments. Scale bars = 25 µm.
